# A Prognostic Model for Prostate Cancer Screened from Stress Granule-Related Genes: An Integrated Bioinformatic Analysis and Experimental Verification of SPAG5 Function

**DOI:** 10.3390/biomedicines14102165

**Published:** 2026-09-24

**Authors:** Zitong Qin, Ting Wang, Runzhang Liu, Pengcheng Chang, Weiping Li

**Affiliations:** 1 The First Clinical Medical College of Lanzhou University, Lanzhou 730000, China; qinzt2024@lzu.edu.cn (Z.Q.);; 2 Department of Urology, The First Hospital of Lanzhou University, Lanzhou 730000, China; 3 Department of Urology, The Second Hospital of Lanzhou University, Lanzhou 730000, China

**Keywords:** prostate cancer, stress granules, risk model, single-cell RNA sequencing, machine learning

## Abstract

**Background:** Stress granules (SGs) are associated with cancer development, but their role in prostate cancer remains unclear. This study aims to identify SG-associated prognostic genes and to discover the biological and clinical significance of these genes. **Methods:** We integrated PCa transcriptomic, clinical, and scRNA-seq data from GEO and TCGA and obtained SG-related genes from GeneCards. Then we applied differential expression analysis, univariate Cox regression, and LASSO regression to identify candidate genes. Subsequently, a risk model was established, encompassing nomogram analysis, functional enrichment, immune infiltration, and scRNA-seq validation. Clinical tissue samples were validated using RT-qPCR and IHC. We performed an shRNA-mediated knockdown of SPAG5 in PC-3 and 22Rv1 cells and then applied RT-qPCR, Western blot, CCK-8, colony formation, wound healing, and Transwell assays to assess its effects. **Results:** Six prognostic genes (SPAG5, FN1, CCK, EGF, SELE, PTGS2) were identified. The risk model classified patients into low-risk and high-risk groups. A nomogram combining risk score and T stage showed moderate discriminatory capacity (AUC > 0.60). High-risk patients exhibited differences in cell cycle pathway enrichment and immune infiltration, and FN1 was positively correlated with Tregs (r = 0.60). ScRNA-seq revealed the dynamic expression of CCK, FN1, and PTGS2 in epithelial cells from paired tumor and normal samples. RT-qPCR and IHC confirmed that EGF and SPAG5 are upregulated in PCa tissue, while CCK, FN1, SELE, and PTGS2 are downregulated. Following SPAG5 knockdown, proliferation, migration, and invasion were inhibited in both cell lines. **Conclusions:** A reliable prognostic model screened from a stress granule-related gene set was established. Experimental data linking this model to the immune microenvironment and epithelial differentiation support the notion that SPAG5 is an oncogenic factor, suggesting that SPAG5 warrants further investigation as a potential prognostic biomarker, though prospective validation is required before clinical translation.

## 1. Introduction

Prostate cancer (PCa) is one of the most prevalent malignant tumors in men globally and is the second leading cause of cancer-related mortality. It is a major factor contributing to the rising male mortality rate globally [1,2]. Globally, it is projected that by 2040, there will be 2.3 million new cases of PCa annually, with approximately 740,000 deaths from the disease, primarily due to population aging and population growth [3]. Epidemiological data indicate a substantial increase in its prevalence among persons over 55 years of age [4]. Currently, common treatments for PCa include surgery, radiotherapy, chemotherapy, and hormone therapy [5]. For in situ prostate cancer, radical prostatectomy combined with radiation therapy, chemotherapy, and other treatments can yield good clinical results, and most patients can be cured [6]. Although during treatment, androgen deprivation therapy (ADT) can significantly reduce testosterone levels, many patients still eventually develop resistance and develop castration-resistant prostate cancer (CRPC) or even metastatic castration-resistant prostate cancer (mCRPC). The 5-year survival rate for these patients is less than 30% [7]. Although therapies such as PARP inhibitors and immunotherapy have advanced, preventing high-risk prostate cancer remains challenging [7]. Therefore, the discovery of novel prognostic markers and targets is important for improving the prognosis of prostate cancer patients.

SGs are membrane-less structures formed via liquid–liquid phase separation (LLPS) in response to various cellular stress conditions [8,9]. SGs play a central role in responding to stress. They can temporarily halt translation, isolate damaged mRNA, and regulate signaling pathways (such as mTOR and p53) to promote cell survival, thereby maintaining cellular homeostasis [10,11]. When tumor cells are exposed to chronic stress (such as malnutrition or hypoxia), SGs form in the tumor microenvironment (TME). SGs are associated with PCa progression, potentially through stabilizing oncogenic mRNAs (such as MYC) and suppressing antitumor immune responses, which may contribute to PCa proliferation, metastasis, drug resistance, and immune evasion [11,12,13]. Single-cell RNA sequencing (scRNA-seq) overcomes averaging effects inherent in traditional bulk RNA-seq by performing transcriptomic sequencing at the single-cell level. It is particularly well-suited for identifying rare cell populations. PCa is a highly heterogeneous disease with multiple stages of progression, making RNA-seq a useful tool for identifying PCa subtypes and monitoring disease progression [14,15].

To identify stress granule-associated prognostic genes in PCa, we leveraged data from the GEO and TCGA databases and conducted a series of analyses comprising differential expression, LASSO regression, and univariate Cox. Using these prognostic genes, we constructed a risk model for PCa. Furthermore, we performed a series of investigations comprising functional enrichment, drug sensitivity analyses, and immune infiltration to characterize how PCa patients stratified into high- versus low-risk categories differ. Concurrently, we conducted single-cell analysis to identify key cell populations within PCa and performed pseudotime analysis. These findings offer valuable insights that may improve the clinical decision-making and prognostic assessment of PCa patients.

## 2. Materials and Methods

### 2.1. Data Collection

To investigate transcriptomic profiles associated with biochemical recurrence (BCR), prostate cancer-related data were obtained from two major public datasets. The primary dataset came from the TCGA-PRAD project via the GDC data portal (https://portal.gdc.cancer.gov/; accessed on 4 March 2025), comprising a total of 483 primary tumor and 51 normal control samples, of which 408 tumors had complete BCR follow-up data. Supplementary expression data were obtained from GEO, including two independent cohorts: GSE70769 (GPL10558 platform), with 94 tumor samples, 92 of which were BCR samples, and GSE220095 (GPL16791 platform), with 176 tumor samples, of which 75 were BCR-related. The GEO datasets were retrieved on 4 March 2025 and 20 January 2026, respectively. Additionally, expression profiles were obtained from GEO scRNA-seq data from the GSE176031 dataset (GPL11154 platform), which included a total of 17 prostate cancer tumor samples and 8 matched non-malignant prostate tissue samples. To facilitate subsequent analysis, we retrieved stress granule-related genes (SGRGs) from the GeneCards database (https://www.genecards.org), yielding 844 candidate genes that met the predefined relevance score threshold of greater than 4 (see Supplementary Table S1). For cross-platform analyses, RNA-seq and microarray data were preprocessed independently according to platform-specific protocols. TCGA-PRAD RNA-seq data were processed as FPKM matrices and log2-transformed (log2 [FPKM + 1]) prior to analysis. Microarray data from GEO datasets (GSE70769 and GSE220095) were log2-transformed if the raw expression value distribution indicated skewness. No direct merging or batch correction was performed across platforms; the validation of the risk model was conducted independently within each dataset using the same risk score formula with fixed LASSO-derived coefficients.

### 2.2. Differential Expression Analysis

To identify DEGs between PCa and normal prostate tissue, we used the DESeq2 algorithm (v1.40.2) to analyze the expression matrix of TCGA-PRAD [16]. We filtered DEGs using a threshold of |log_2_FC| > 1.0 and adjusted *p* < 0.05. The resulting DEG profile was visualized using a volcano plot (ggplot2 package, version 3.5.1), and we visualized the results of unsupervised hierarchical clustering as a heatmap using the pheatmap package (version 1.0.12) [17,18]. These graphical representations effectively highlight significant DEGs and their expression patterns across different samples.

### 2.3. Identification and Function Analyses of Candidate Genes

To prioritize candidate genes for downstream functional investigation, we first identified the intersection between the DEG list and the SG-RG collection using the ggvenn package (v0.1.10), which generated a set of overlapping genes [19]. Subsequently, the functional annotation of this candidate gene set was carried out via clusterProfiler (v4.15.0.003), encompassing both GO terms and KEGG pathway enrichment analyses (*p* < 0.05) [20]. Separately, a PPI network was reconstructed for the overlapping genes using the STRING database (https://string-db.org/), with a minimum required interaction score of >0.70. Isolated nodes without any connections were then removed [20,21].

### 2.4. Determination of Prognostic Genes

To initially identify prognosis-associated genes in the TCGA-PRAD cohort, we performed univariate Cox proportional hazards regression via the survival R package (version 3.7-0), applying the prespecified inclusion criteria of a hazard ratio (HR) deviating from 1.0 and a nominal *p*-value < 0.05. The underlying proportional hazards assumption for each candidate was concurrently verified (*p* > 0.05) [22]. To further refine this gene set and mitigate overfitting, we implemented LASSO-penalized Cox regression using the glmnet package (v 4.1-8), with the optimal penalty parameter (lambda.min) selected via 10-fold cross-validation for feature selection [23]. Finally, we reanalyzed the differential expression of the prognostic genes selected by LASSO using the Wilcoxon rank-sum test (*p* < 0.05).

### 2.5. Establishment and Assessment of Risk Model

Based on the prognostic genes identified via the risk model, the risk score was calculated using the following formula:

$$\mathrm{risk}\mathrm{score}=\sum_{i=1}^{n}\left(\mathrm{coefi}*\mathrm{Xi}\right)$$

Here, “coef” represents coefficients for the biomarker obtained using the LASSO method, and “X” represents its expression level. The optimal cutoff value for risk score was determined using the surv_cutpoint function (survminer package) with the parameter minprop = 0.4, which ensures that each risk group contains at least 40% of the total samples to avoid subgroups with insufficient sample sizes. Of note, this 0.4 value defines the minimum proportion of samples per group and does not represent any expression level cutoff. Based on the optimal cutoff value, patients with incomplete clinical data were excluded, and only tumor and normal tissue samples from the initial diagnosis were retained for the 408 TCGA-PRAD patients. This resulted in an HRG and LRG. The distribution of risk scores is represented by a risk curve. The survival status of each patient group is illustrated using scatter plots. And the prognostic value of risk stratification was assessed using Kaplan–Meier survival curves. The overall survival curve was plotted using functions from the survminer package (v 0.4.9; *p* < 0.05). To quantitatively evaluate predictive performance, the timeROC package (v 0.4) was used to perform time-dependent ROC analysis, examining predictive accuracy over three time periods: 1 year, 3 years, and 5 years. The AUC was calculated for each period to measure the predictive accuracy of the features [24]. Additionally, the discriminative ability of the risk model was assessed using Harrell‘s concordance index (C-index), calculated with the concordance function in the R package survival (v 3.7-0) based on Surv(BCR.time, BCR). The 95% confidence intervals (CIs) for the C-index were estimated using 1000 bootstrap resamples. To evaluate the model’s generalization ability and stability, the analysis workflow was replicated using two external datasets (GSE70769 and GSE220095). The same procedures and methodological steps were strictly followed to ensure that the results were comparable and reproducible.

### 2.6. Independent Prognostic Analysis

To evaluate whether the LASSO-derived risk signature functioned as an independent prognostic indicator, we conducted univariate and multivariate Cox regression analyses on the TCGA-PRAD cohort using the survival package (v 3.7-0). Covariates were considered with HR ≠ 1 and *p* < 0.05, and the PH assumption was simultaneously tested and satisfied for all included variables (*p* > 0.05). We subsequently constructed a prognostic nomogram via the rms package (v 6.8-1), which was designed to graphically predict the probabilities of biochemical recurrence (BCR)-free survival at 1-, 3-, and 5-year post-treatment intervals [25]. Calibration curves were generated using regplot (v 1.1) to evaluate model predictive accuracy [26]. In order to provide further support for this study’s findings, additional univariate and multivariable Cox regression analyses were conducted on validation sets (GSE70769 and GSE220095). These analyses assessed the independent and joint prognostic value of candidate variables across the independent cohorts. To evaluate the incremental prognostic value of the risk score beyond conventional clinicopathological factors, a clinical Cox model containing only the Gleason score was compared with a combined model incorporating both the Gleason score and the risk score in the same patient subsets with complete data. Model discrimination was assessed using Harrell’s C-index, and model fit was compared using likelihood ratio tests. For external validation, model coefficients fixed from the TCGA-PRAD training set were directly applied without re-estimation.

### 2.7. Clinical Characteristic Analysis and GSEA

To further explore risk score differences between the HRG and LRG across clinical subgroups, Wilcoxon (two groups) and Kruskal–Wallis tests (multiple groups) were used (*p* < 0.05). We applied Spearman’s correlation to evaluate associations between risk score and clinical features, with |cor| > 0.30 and *p* < 0.05 considered significant.

We performed functional enrichment analysis to explore biological functions associated with the HRG and LRG. Differential expression between the two groups was first analyzed using DESeq2 (v3.4.1), and DEGs were ranked by log_2_FC values. Given that SGs facilitate tumor progression through the modulation of the immune microenvironment [27], we sought to explore their potential link to immune evasion via functional enrichment profiling. To contextualize the biological relevance of the SG-associated gene signatures, we performed GSEA using the clusterProfiler package, with reference to KEGG pathway gene sets (c2.cp.kegg.v2022.1.Hs.symbols.gmt) from MSigDB (v 2022.1). Gene lists were ranked according to their differential expression signals prior to enrichment testing. Significance was set at |NES| > 1.0, FDR < 0.25, and nominal *p* < 0.05.

### 2.8. Immune Infiltration and Immune Microenvironment Analyses

The abundance of tumor-infiltrating immune cells across 28 distinct cellular phenotypes in both the HRG and LRG was quantified using the ssGSEA approach implemented within the “GSVA” bioinformatics package (v 1.53.28) [28,29]. Subsequently, non-parametric Wilcoxon rank-sum statistical testing was implemented to assess comparative abundance variations in immune infiltration patterns using nominal *p*-values. To adjust for multiple comparisons across 28 immune cell types, Benjamini–Hochberg false discovery rate (FDR) correction was further applied. Cell types with an FDR < 0.05 after correction were regarded as statistically significantly different and retained for subsequent downstream analyses. We identified immune cell subpopulations with significantly disparate infiltration levels between groups, which were retained for subsequent analyses. The interrelationships among these differentially infiltrated cell types, as well as their associations with both the risk score and the prognostic gene signature, were then evaluated through correlation analysis (|cor| > 0.30 and *p* < 0.05). Among the 79 predefined immune checkpoint genes [30], 12 genes were absent from our expression matrix; Wilcoxon rank-sum tests were performed on the remaining 67 ICGs. BH-FDR correction was applied over these 67 tests, and genes with an FDR < 0.05 were considered statistically significant.

### 2.9. Somatic Mutation and Drug Sensitivity Analyses

Somatic mutation landscapes were characterized for HRG and LRG patients using the maftools package (v2.16.0) [31]. TMB was then estimated for each sample, and its distribution was compared between the two risk cohorts using the Wilcoxon test (*p* < 0.05). To further explore the combined effect of risk stratification and mutational load on patient outcomes, we stratified the cohort into high- and low-TMB subsets and subsequently performed Kaplan–Meier survival analysis (survminer v0.4.9) across four combinatorial groups (high-TMB + HRG, high-TMB + LRG, low-TMB + HRG, and low-TMB + LRG).

We retrieved half-maximal inhibitory concentration (IC50) data for 274 compounds from the GDSC repository (https://www.cancerrxgene.org/) and estimated their values for each PCa sample using the pRRophetic algorithm (v0.5.0) [32]. Differences in the estimated IC50 values between the HRG and LRG were subsequently assessed using the Wilcoxon test (*p* < 0.05).

### 2.10. scRNA-Seq Analysis

scRNA-seq analysis can investigate whether such genes functionally interact with stress granules (SGs), thereby facilitating the phenotypic characterization of SG-associated cell types and identifying SG-associated cell populations in prostate cancer (PCa) tissue. Although SGs are transient molecular assemblies, the gene expression programs they regulate can persist in tumor cells. Therefore, scRNA-seq can capture their downstream molecular signals. The GSE176031 dataset was analyzed using a standard single-cell RNA sequencing workflow with Seurat version 5.1.0 [33]. GSM samples were merged into a Seurat object with orig.ident retained to track sample origin, and patient identities were matched based on GEO metadata. For group comparisons, data from the same patient were aggregated at the patient level prior to statistical testing to control for intrapatient correlation. No additional batch correction methods (e.g., Harmony or CCA) were applied beyond the standard Seurat workflow; instead, original sample identities were preserved to allow for the tracking of potential intersample technical variability. Three methods were used to initially exclude low-quality cells: those with 300–3000 genes, 500–8000 UMIs, and a mitochondrial gene content less than 20%. Subsequently, the filtered data underwent log-normalization, and HVGs across cells were identified using a VST method. These HVGs were then used as input for PCA, with the optimal number of principal components determined via the JackStraw procedure. For cluster detection, we constructed a shared nearest-neighbor graph and applied the Louvain algorithm at a resolution of 1, with the resulting clusters projected onto a two-dimensional UMAP embedding. Differential expression testing across clusters was performed via FindAllMarkers to obtain cluster-specific gene sets, which were subsequently used for cell type annotation with reference to established marker signatures from the literature [34]. Cell type annotation was then performed by combining these cluster-specific genes with established canonical markers from the literature [34]: epithelial cells (ACPP, MSMB, PLA2G2A), T cells (CCL5, CD96, CD2), myeloid cells (IL1B, IGSF6, MSR1, C1QC), endothelial cells (ELTD1, SELE, ECSCR), smooth muscle cells (SOX17, MYH11, PLN), fibroblasts (CASQ2, DCN), mast cells (CPA3, TPSAB1, HDC), and B/plasma cells (MS4A2, IGHM, CD79A).

Cell abundance was compared by the Wilcoxon test (*p* < 0.05), and those with significant differences were defined as differential cells. Of note, individual cells derived from the same patient are statistically non-independent. In this work, individual cells were used for cell type annotation, visualization and descriptive exploration of cellular heterogeneity. Patients were treated as independent biological units for group-level comparisons to avoid pseudoreplication caused by within-patient cell correlation. All statistical inferences were performed at the patient level rather than at the single-cell level. Functional analysis was applied using ReactomeGSA (v1.14.0) [35].

### 2.11. Identification of Key Cells and Pseudo-Temporal Analyses

A statistical evaluation of survival-related genetic expression patterns across prostate cancer specimens versus normal tissue samples was conducted through non-parametric Wilcoxon rank-sum testing, utilizing a statistical threshold of *p* < 0.05. Cells with obvious expression differences were defined as key cells. To further analyze these key cells, secondary dimensionality reduction was performed using the FindNeighbors and FindClusters functions (resolution = 0.3). This approach identified differential subclusters within the key cells (inclusion required gene expression in ≥50% of cells and |log_2_FC| ≥ 0.25 (approximately 1.19-fold)). We performed KEGG functional pathway enrichment analysis using the clusterProfiler package (v 4.15.0.003) to systematically identify biological pathways that were significantly overexpressed (*p* < 0.05) in various cell subpopulations.

To investigate the differentiation trajectories and transcriptional dynamics of core cell subsets, the Monocle (2.28.0) [36] algorithm was used. Of note, the root of the trajectory was not manually predefined but determined by the orderCells function based on data-driven transcriptional similarity. Therefore, the inferred root serves as a reference point for defining the analysis direction rather than a validated biological starting state. Pseudotime trajectory analysis is a descriptive visual approach and was not used for inferential statistical testing at the single-cell level.

### 2.12. Cell Culture

The HEK-293, PC-3, and 22Rv1 used in this study were provided by Shanghai Jinyuan Biotechnology Co., Ltd. (Shanghai, China; JY757, JY185). For the routine culture of PC-3 and 22Rv1 cells, RPMI-1640 medium (Gibco, Grand Island, NY, USA) was used, while HEK-293 cells were cultured in DMEM (Gibco). Media were supplemented with 10% FBS and 100 μg/mL PS. All cell types were cultured in a fully humidified environment at 37℃, 5% CO_2_. All cell lines used in the experiments were in the logarithmic growth phase.

### 2.13. RT-qPCR

Ten matched pairs of PCa and adjacent nontumor tissues were collected at First Hospital of Lanzhou University between 2024 and 2025, with all procedures authorized by the institutional ethics committee (LDYYLL-2025-895). Total RNA was isolated from both the clinical specimens and the PC-3/22Rv1 cell lines using TRIzol reagent (Ambion, Austin, TX, USA), after which cDNA was synthesized with the SureScript cDNA Kit (Servicebio, Wuhan, China). The amplification step was performed on a SYBR Green platform (Servicebio) under initial denaturation at 95 °C for 30 s, followed by 40 cycles of 95 °C for 5 s and 60 °C for 30 s. Transcript levels for each target gene were calculated via the 2^−ΔΔCt^ method with GAPDH as the internal reference, and the primer sequences used are listed in the accompanying table. Additionally, GraphPad Prism (version 5.0.0) was used for data plotting and *p*-value calculations [36].

### 2.14. Immunohistochemistry (IHC)

Immunohistochemical staining was performed on paraffin-embedded tissue sections. After standard dewaxing and rehydration, the sections were placed in a buffer containing citrate or EDTA and heated to perform antigen retrieval. To reduce nonspecific binding, hydrogen peroxide was first applied to inactivate it, followed by blocking with normal serum. The slices were then incubated at 4 °C overnight with the appropriate antibody. The next day, an HRP-labeled secondary antibody was added to the sample, and immunoreactivity was visualized using a DAB substrate. The nuclei were subsequently stained with a pale nuclear stain. Finally, the stained sections were sequentially dehydrated with a series of ethanol solutions, dewaxed with a clearing agent, and embedded in cedar oil and covered with a coverslip for microscopic examination [37]. The antibody information used is as follows: SPAG5 (Proteintech, Rosemont, IL, USA; 14726-1-AP, 1:400), FN1 (Proteintech, 15613-1-AP, 1:4000), CCK (Proteintech, 13074-2-AP, 1:200), EGF (Proteintech, 27141-1-AP, 1:400), SELE (Proteintech, 20894-1-AP, 1:4000), and PTGS2 (Proteintech, 27308-1-AP, 1:4000).

### 2.15. shRNA Knockdown of SPAG5

To stably suppress SPAG5 expression, three different short hairpin RNAs (shRNAs) targeting SPAG5 (sh1, sh2, sh3) were designed, and a non-targeting NC was included. All constructs were cloned into the pLent-U6-shRNA-CMV-GFP-P2A-puromycin vector. Lentiviral particles were generated by co-transfecting HEK293T cells with shRNA-expressing plasmids and packaging plasmids using Lipofectamine 3000. Viral supernatants were collected at 48 and 72 h and filtered to remove cell debris. The filtered viral particles were incubated with Target PC-3 and 22Rv1 cells, with the addition of 8 μg/mL polybrene. After infection, cells were cultured for an additional 24 h, followed by 7–10 days of selection in medium containing 2 μg/mL puromycin.

### 2.16. Western Blot (WB)

For Western blot analysis, monolayer-cultured PC-3 and 22Rv1 cells were harvested and lysed with RIPA buffer (Beyotime, Shanghai, China; P0013B) supplemented with a mixture of protease and phosphatase inhibitors (Beyotime P1046). Precipitates were removed from the cell extracts by centrifugation, and the protein concentration in the supernatant was determined using the BCA protein assay (Solarbio, Beijing, China; PC0020). Equal amounts of protein were separated by SDS-PAGE and subsequently transferred to a PVDF membrane (Thermo Fisher, Waltham, MA, USA; 88518). To minimize nonspecific antibody binding, the membrane was blocked with TBST and 0.1% Tween 20 and a 5% solution of skim milk powder or bovine serum albumin (BSA) at room temperature for 1 h. Subsequently, the membrane was incubated at 4 °C overnight with the SPAG5 antibody (1:1000, Proteintech, 14726-1-AP) and the GAPDH antibody (1:5000, Proteintech, 60004-1-Ig) at 4 °C. The membrane was incubated in the HRP-labeled secondary antibody (1:5000) for one hour. Immunoreactive bands were detected using an ECL substrate (Solarbio, PE0010). Analysis was performed using ImageJ software (version 1.54r).

### 2.17. CCK-8 Assay

PC-3 and 22Rv1 cell lines were seeded into 96-well plates at a density of 2 × 10^3^ cells per well for 24 h. Then, 10 μL of CCK-8 reagent (YEASEN, Shanghai, China; Product No. 40203ES76) was added at the time points of 0, 24, 48, and 72 h. After incubating at 37 °C for 2 h, optical density (OD) values were measured at 450 nm. Relative cell growth was calculated as the percentage of OD values normalized to the 0 h baseline of each respective group.

### 2.18. Wound Healing Assay

PC-3 and 22Rv1 cells were grown in six-well plates to approximately 90% confluence. Next, using sterile 200 μL pipette tips, a straight line was drawn across each well. Cell debris generated by this scraping process was washed away with PBS. Thereafter, all cells were cultured in serum-free medium until the end of the experiment to avoid the influence of serum on cell proliferation.

### 2.19. Transwell Assay

Corning Transwell chambers with an 8 μm pore size (Corning, NY, USA; 3422) were pre-coated with a 1:8 dilution of Matrigel (Corning, 354234), and the assembly was incubated at 37 °C for one hour to establish a reconstituted basement membrane. PC3 cells (2 × 10^4^) or 22Rv1 cells (1 × 10^5^) were seeded into the upper chamber, while the lower chamber was filled with complete medium, which served as the chemical attractant. After 24 h, the cells migrated through the membrane and attached to the lower surface. They were stained with 0.1% crystal violet.

### 2.20. Colony Formation Assay

PC-3 and 22Rv1 cells were seeded 500 cells per well in 6-well plates and cultured for 10 days. Fresh medium was added every three days during the culture period. The colonies were gently rinsed with PBS, fixed with 4% PFA for 15 min, then stained with 0.5% crystal violet.

### 2.21. Statistical Analysis

All statistical analyses were performed using R (v 4.2.3), with intergroup comparisons evaluated by the Wilcoxon test (*p* < 0.05).

## 3. Results

### 3.1. Determination and Function Exploration of 126 Candidate Genes

Differential expression analysis revealed a total of 2981 DEGs between PCa tumors and normal tissues, of which 1265 exhibited upregulation and 1716 downregulation (volcano and heatmap representations are provided in Figure 1A,B). When these DEGs were overlapped with the SG RG inventory (844 genes), a set of 126 overlapping candidates was obtained for subsequent investigation (Figure 1C).

Enrichment analyses were conducted to characterize the functional properties of 126 candidate genes, with the output summarized (Figure 1D,E). For GO annotation, the candidates showed statistically significant enrichment across multiple ontologies (*p* < 0.05), with the top-ranked terms (based on *p*-values) in the three main categories represented by “regulation of monoatomic ion transport” for biological processes, “secretory granule lumen” for cellular components, and “hormone activity” for molecular functions; the top eight terms within each ontology are detailed in Figure 1D. Parallel KEGG pathway analysis identified the significant overrepresentation of the candidate genes in a range of pathways (*p* < 0.05), with “vascular smooth muscle contraction”, “renin secretion”, and “complement and coagulation cascades” being among the 15 most significant entries (Figure 1E).

The PPI network for the 126 genes revealed their interconnections (Figure 1F). Of particular interest, ALB showed marked interaction connectivity with F2, CRP, and AGT, pointing to the possibility that ALB and its associated partners may contribute critically to the pathogenesis of PCa and could serve as actionable therapeutic targets.

### 3.2. Construction of Well-Performing Risk Model

From 126 genes, 13 survival-related genes were identified (Figure 2A and Figure S1). The LASSO method then narrowed the selection down further to six prognostic genes (lambda.min = 0.006262275) (Figure 2B). Notably, CCK, FN1, PTGS2, and SELE showed significantly higher expression in control samples, while EGF and SPAG5 were notably more expressed in PCa samples (Figure 2C).

A prognostic risk model was subsequently established incorporating six genes: SPAG5, FN1, CCK, EGF, SELE, and PTGS2. The risk score was defined by the linear combination of these genes weighted by their respective LASSO-derived coefficients, as follows: 0.474 × SPAG5 + 0.286 × FN1 − 0.106 × CCK − 0.291 × EGF − 0.166 × SELE − 0.070 × PTGS2. Under the sample proportion constraint of “minprop = 0.4”, the optimal risk score cutoff value of 0.7538191 was calculated in the TCGA-PRAD cohort (*n* = 408), which stratified patients into an HRG (*n* = 167) and LRG (*n* = 241). Applying the same “minprop = 0.4” constraint in the GSE70769 cohort, we re-estimated the cohort-specific risk score cutoff and obtained 41 HRG and 51 LRG patients. This risk stratification substantially separated the two prognostic subgroups, with the HRG experiencing considerably poorer survival outcomes (Figure 2D,E), and the Kaplan–Meier survival estimates demonstrated a marked divergence between the two risk groups in both TCGA PRAD (*p* < 0.0001) and GSE70769 (*p* = 0.0022) (Figure 2F,G). ROC analysis showed AUC values exceeding 0.60 across both datasets, indicating moderate discriminatory capacity for BCR-free survival (Figure 2H,I). In the TCGA-PRAD training cohort (*n* = 408), 49 patients experienced BCR, yielding an event rate of 12.01%. The C-index of the risk model in this cohort was 0.706 (95% CI: 0.632–0.780). In the GSE70769 validation cohort (*n* = 92), 45 BCR events were observed (event rate: 48.91%), with a C-index of 0.639 (95% CI: 0.547–0.732). In the GSE220095 validation cohort (*n* = 176), 75 BCR events occurred (event rate: 42.61%), with a C-index of 0.597 (95% CI: 0.529–0.665) (Supplementary Table S2). To evaluate predictive ability, this model was also applied to the independent GSE220095 dataset, which contains 176 samples, of which 75 are recurrent, and 101 are non-recurrent. With the fixed LASSO gene coefficients and the identical “minprop = 0.4” constraint, a cohort-specific risk score cutoff was re-estimated for GSE220095 to classify patients into high-risk (*n* = 75) and low-risk (*n* = 101) groups. The risk map (Figure 2J) shows differences in the expression profiles of six genes between the two subgroups. The Kaplan–Meier survival curves indicate a significant difference in prognosis between the HRG and LRG (*p* = 0.0013; Figure 2K). Furthermore, the risk score demonstrates good predictive ability, with an AUC value ranging from 0.6 to 1.0 (Figure 2L). Therefore, the newly developed prognostic score may assist in stratifying PCa patients, though its clinical utility requires further validation in larger independent cohorts.

### 3.3. Establishment of Good Nomogram Based on Independent Prognostic Factors

Univariate Cox regression analysis showed that risk score, T stage, Gleason score, and N stage were significantly associated with BCR-free survival (Figure 3A,E and Figure S2). In multivariate analysis, T stage and Gleason score remained independent prognostic factors, whereas the risk score did not reach statistical significance in the GSE220095 validation cohort (*p* = 0.642; Figure 3F). This suggests that the prognostic information carried by the risk score partially overlaps with conventional clinicopathological factors and that its independent effect was attenuated in this external cohort. It should be emphasized that the risk score was predefined and fixed from the training cohort rather than re-selected in the validation cohort. Based on these findings, T stage and Gleason score were incorporated as independent prognostic factors to develop a nomogram for predicting BCR-free survival. Given its significant prognostic value in univariate analysis and its potential to provide additional predictive information, the risk score was also retained as a component of the nomogram. In this nomogram, each variable is assigned a weighted score to reflect its overall importance, and these scores are summed to yield a total score; a higher score indicates a lower probability of BCR-free survival (Figure 3C,G). Calibration plots were used to assess the accuracy of the nomogram in predicting BCR-free survival (Figure 3D,H), showing acceptable predictive capacity with potential value for individualized risk assessment alongside standard clinicopathological factors. To assess the incremental prognostic value of the risk score beyond the Gleason score, the C-index of a clinical model (Gleason score only) was compared with that of a combined model (Gleason score + risk score). In the TCGA-PRAD training cohort (*n* = 408, 49 BCR events), the C-index increased from 0.655 (95% CI: 0.585–0.724) to 0.720 (95% CI: 0.652–0.788), with a ΔC-index of 0.066 (likelihood ratio test: χ^2^ = 12.523, *p* = 0.000402). In the GSE70769 validation cohort (*n* = 88, 45 BCR events), the C-index increased from 0.693 (95% CI: 0.629–0.757) to 0.734 (95% CI: 0.657–0.810), with a ΔC-index of 0.041. In the GSE220095 validation cohort (*n* = 176, 75 BCR events), the C-index was 0.662 (95% CI: 0.616–0.708) for the clinical model and 0.661 (95% CI: 0.598–0.723) for the combined model (ΔC-index = −0.001). Collectively, the risk score improved discrimination over the Gleason score alone in the TCGA-PRAD and GSE70769 cohorts, but this incremental effect was not replicated in GSE220095, suggesting potential cohort heterogeneity in its added predictive value (Supplementary Table S3).

### 3.4. Risk Assessment of PCa Based on Clinical Characteristics

The proportion of differential clinical characteristics across subgroups was analyzed. N0-stage patients exhibited a higher proportion compared to N1-stage patients. Similarly, T3-stage patients had a higher proportion than T2-stage ones, and patients aged >60 years had a higher proportion in both the HRG and LRG (Figure 4A). Regarding Gleason scores, Gleason 7 had a higher proportion in the LRG (63.1%), while Gleason 9 had a higher proportion in the HRG (46%) (Figure 4A).

Further analysis employing the Wilcoxon test demonstrated a significantly higher risk score in patients aged >60 years relative to their younger counterparts (≤60 years) (*p* = 0.042). Additionally, patients in the N1 stage exhibited a significantly higher risk score compared to N0-stage patients (*p* < 0.001), and those in the T3 stage had a notably higher risk score compared to those in the T2 stage (Figure 4B). Furthermore, risk scores differed significantly across Gleason score groups (*p* < 0.05), notably between Gleason 6 and Gleason 7 (*p* < 0.001; Figure 4B).

In conclusion, Spearman correlation analysis revealed a significant positive correlation between risk score and Gleason score (Figure 4C). These results indicate that clinical indicators are closely linked to risk stratification, which is significant for the future development of more personalized treatment.

### 3.5. Exploration of Pathway, Immune Infiltration, and Immune Checkpoint Expression Differences Between HRG and LRG Patients

Compared with the LRG, cell cycle-related pathways were upregulated in the HRG, while pathways relevant to metabolism and contractile function were downregulated (Figure 5A). A total of 33 pathways in the HRG showed significant changes, among which the pathways with the most pronounced enrichment included “cell cycle”, “oocyte meiosis”, “pentose and glucuronate interconversions”, “porphyrin and chlorophyll metabolism”, and “primary immunodeficiency”. Conversely, the pathways most notably downregulated encompassed “cardiac muscle contraction,” “hypertrophic cardiomyopathy (HCM)”, “olfactory transduction”, “arginine and proline metabolism”, and “tight junction” (Supplementary Table S4).

Moreover, immune infiltration scores for 28 distinct immune cell subsets within the HRG and LRG were depicted (Figure 5B). A comparative analysis revealed significant differences in the infiltration levels of 14 immune cell types at a nominal *p* < 0.05 (Figure 5C); after BH-FDR correction for all 28 cell type comparisons, 12 cell types remained significant (FDR < 0.05). For example, activated CD4^+^ T cells were enriched in the HRG (*p* < 0.01), whereas activated B cells exhibited greater infiltration in the LRG (*p* < 0.05), collectively suggesting divergent immune response profiles across the risk categories. Further analysis using Spearman’s correlation coefficient revealed positive correlations between most types of infiltrating immune cells, with particularly strong correlation observed between regulatory T cells and central memory CD8+ T cells (Figure 5D). In addition, the level of infiltration of these immune cell subsets is closely associated with the expression of prognostic genes (Figure 5E). Notably, FN1 expression demonstrated a positive correlation with the infiltration of regulatory T cells.

Of the initial 79 immune checkpoint genes, 12 were missing from the expression matrix. Among the remaining 67 tested genes, 34 showed a nominal *p* < 0.05, and 29 remained significant after BH-FDR correction (FDR < 0.05) (Supplementary Figure S2). CD276 expression was elevated in the HRG (*p* < 0.01), and CEACAM1 expression was higher in the LRG (Figure 5F). Overall, different ICG expression patterns may enable risk stratification and serve as targets for immunotherapy, making them predictive biomarkers.

### 3.6. Comprehensive Analyses of Somatic Mutations and Drug Sensitivity in HRG and LRG Patients

A mutational profile analysis was performed on samples from the HRG and the LRG, revealing that TP53, TTN, and SPOP were the three most frequently mutated genes (Figure 6A,B). Most of the detected alterations were missense mutations and multi-hit events, which is consistent with the known fact that these two genes play important roles in defining the phenotypic characteristics of the two risk subgroups. In addition, patients classified as in the HRG had significantly higher TMB values than those classified as in the LRG (Figure 6C). Kaplan–Meier survival analysis shows that patients with low TMB have a better survival prognosis (*p* = 0.019; Figure 6D). Based on the risk stratification, there were significant differences in prognosis among the four patient groups: high-risk patients had a poor prognosis (*p* < 0.0001), and the high-risk TMB/HRG group had the poorest survival rate (Figure 6E). These results indicate that an increased TMB, when combined with HRG status, is associated with a higher likelihood of poor prognosis.

The drug susceptibility profile indicates that there are nine different compounds for which predicted IC50 values were estimated by the pRRophetic algorithm for the HRG and LRG (Figure 6F). For instance, patients in the HRG had significantly higher predicted IC50 values for temsirolimus (*p* < 0.0001), while patients in the LRG showed elevated predicted IC50 values for vinblastine (*p* = 0.00659; Figure 6F). Of note, these IC50 values are computationally derived predictions based on gene expression profiles, rather than experimentally measured drug responses. Therefore, they should be interpreted as in silico estimates rather than direct indicators of clinical treatment efficacy.

### 3.7. Ascertainment of Eight Annotated Cells

After excluding ineligible cells and genes, the remaining 13,911 cells and 21,334 genes were used for analysis, and 2000 HVGs were selected from among them (Supplementary Figure S3A,B). The top 30 principal components identified by PCA that were statistically significant (*p* < 0.05) were used for subsequent analysis (Supplementary Figure S3C). UMAP delineated the cells into 18 distinct clusters (Figure 7A). The subsequent cell type classification of the resulting clusters identified eight major cell types: T cells, epithelial cells, myeloid cells, endothelial cells, smooth muscle cells, fibroblasts, mast cells, and B/plasma cells (Figure 7B,C). It is evident that both PCa and normal samples are predominantly composed of epithelial cells (annotated based on canonical marker genes) (Figure 7D). A comparison of cell type proportions revealed statistically significant differences in epithelial cells, monocytes, and T cells among the different groups (Figure 7E). Functional enrichment analysis showed the “melanin biosynthesis” pathway is primarily associated with epithelial cells and medullary cells (Figure 7F).

### 3.8. Identification and Exploration of Differentiation Trajectories of Key Cells

UMAP shows the distribution of labeled cell clusters based on gene expression (Figure 8A). Importantly, the Wilcoxon rank-sum test revealed that PCa samples exhibit significantly different CCK expression levels between normal and cancerous tissues (*p* < 0.001; Supplementary Figure S4), suggesting that CCK can serve as a biomarker for distinguishing between normal and tumor tissues. Since the primary cell type in PCa is epithelial, subsequent dimensionality reduction and clustering analysis further divided the epithelial cells into six distinct subpopulations (Figure 8B), with Subcluster 0 representing the largest fraction (29.4%) (Figure 8C). KEGG pathway enrichment analysis revealed distinct biological pathways associated with different epithelial subclusters (Figure 8D). Specifically, Subcluster 0 was enriched in the phagosome pathway; Subcluster 2 exhibited enrichment in the chemokine signaling pathway; Subcluster 3 showed enrichment in viral protein–cytokine receptor interaction; Subcluster 4 displayed enrichment in hematopoietic cell lineage pathways; and Subcluster 5 was characterized by enrichment in necroptosis. Of note, while these pathway enrichments suggest potential functional differences among subclusters, they should be interpreted with caution. In particular, the presence of hematopoietic/immune-related pathway enrichment in some epithelial subclusters does not necessarily indicate contamination or misannotation, as tumor-associated epithelial cells can upregulate immune-related transcriptional programs in the tumor microenvironment. Nevertheless, the possibility of doublets or ambient RNA contamination cannot be entirely excluded. Therefore, these observations are presented as exploratory findings requiring further experimental validation.

Pseudotime trajectory analysis was performed to explore the transcriptional continuum among epithelial subclusters, ordering cells along a pseudotime axis based on transcriptional similarity. The root of the trajectory was defined based on the transcriptional characteristics of the corresponding cell group and its relative position within the trajectory, serving as a reference point to define the analysis direction rather than a validated biological starting state. The inferred ordering followed approximately Subcluster 2 → Subcluster 3 → Subcluster 1 → Subcluster 5 → Subcluster 4 → Subcluster 0. Of note, this pseudotime ordering reflects relative transcriptional states and is presented as exploratory evidence, rather than evidence of a true temporal sequence, epithelial differentiation process, or tumor progression direction (Figure 8E,F). The pseudotime analysis can reveal the dynamic changes in the expression of relevant genes during epithelial differentiation. Specifically, FN1 and PTGS2 are highly expressed during early differentiation, suggesting that they may play a role in the initial transformation of epithelial cells into tumor-associated cell populations and are also essential for establishing a supportive TME. Conversely, the expression of CCK increases in later stages, suggesting that it is involved in high-level differentiation processes and the development of cancer. Elucidating these differences in temporal expression will help us to clarify the mechanisms underlying tumorigenesis and development and identify targets for early intervention.

### 3.9. Expression Analysis of Prognostic Genes

Consistent with bioinformatic predictions, RT-qPCR validation shows that PCa tissues exhibited significantly increased expression levels of EGF and SPAG5, while CCK, FN1, SELE, and PTGS2 showed markedly decreased levels compared with normal control tissues (*p* < 0.05). Notably, the differential expression of FN1, SELE, and PTGS2 was especially pronounced (Figure 9A). Immunohistochemical (IHC) analysis further confirmed the altered protein expression of SPAG5, FN1, CCK, EGF, SELE, and PTGS2, revealing significant differences between tumor tissues and adjacent normal counterparts. Furthermore, IHC analysis showed the altered protein expression of SPAG5, FN1, CCK, EGF, SELE, and PTGS2 between tumor tissues and adjacent normal counterparts in representative cases, but no quantitative or prognostic association analysis was performed due to limited sample size (Figure 9B).

### 3.10. SPAG5 Knockdown Suppresses Prostate Cancer Cells

To explore the functional role of SPAG5 in PCa, we established stable SPAG5 knockdown PC 3 and 22Rv1 cell lines via three independent short hairpin RNA constructs (shSPAG5 1, shSPAG5 2, and shSPAG5 3), alongside negative and blank controls. RT qPCR and Western blot analyses validated marked decreases in both SPAG5 mRNA and protein levels in all knockdown groups relative to the controls (Figure 10A–D). Among these, shSPAG5 1 and shSPAG5 2 showed the most potent knockdown efficacy and were thus chosen for all subsequent functional experiments.

CCK 8 and colony formation assays were employed to evaluate the proliferative capacity of SPAG5 knockdown cells. Colony formation was significantly inhibited in the knockdown groups, with the degree of suppression positively correlating with the efficiency of gene knockdown (Figure 10E-H). SPAG5 knockdown markedly decreased relative cell growth in a time-dependent manner, with the growth curves of both the shSPAG5 1 and shSPAG5 2 groups remaining consistently below those of the control and NC groups (Figure 10I,J). These results indicate that SPAG5 plays a crucial role in sustaining proliferative capacity.

Wound healing and Transwell assays were performed to evaluate the migratory and invasive abilities of cells with SPAG5 knockdown. Compared with the control group, the SPAG5 knockdown group showed a significant reduction in the number of migrating cells, as well as a marked impairment in cell migration and invasion capabilities (Figure 11A–H).

These findings indicate that knocking down SPAG5 can inhibit the proliferation, migration, and invasion of PCa cells, suggesting that SPAG5 may be a tumor-promoting factor that drives the progression of prostate cancer.

## 4. Discussion

SGs are dynamic, membrane-less organelles that form within cells in response to environmental stimuli. They help cells survive under stress by sequestering messenger RNA (mRNA) with stalled translation and signaling proteins [38]. The assembly of stress granules is triggered by microenvironmental conditions such as hypoxia and oxidative stress within the TME. However, the underlying mechanisms remain to be elucidated [39]. In PCa, SGs function as critical stress survival hubs and are intimately linked to tumor progression. We identified six prognostic genes in PCa, including SPAG5, FN1, CCK, EGF, SELE, and PTGS2, as well as a pivotal cellular subset, epithelial cells. Utilizing these six prognostic genes, we developed a predictive model with substantial prognostic accuracy. Subsequently, through analyses of gene enrichment, immune infiltration, and drug sensitivity, we provided new insights into the prognosis and treatment of PCa. In addition to the above, single-cell transcriptomic analysis was used to elucidate mechanisms at the cellular level, providing a multidimensional framework to support future mechanistic studies. Notably, our findings revealed that patients classified as in the HRG exhibited significantly higher predicted IC_50_ values for temsirolimus based on the pRRophetic algorithm. However, these values are computationally derived predictions rather than measured drug responses, and the underlying mechanisms remain speculative. Therefore, these observations should not be interpreted as clinical evidence of resistance. Firstly, SG-positive cells could utilize eIF2α-ATF4-mediated adaptive signaling pathways to increase the expression of pro-survival proteins, thereby attenuating cell death signals induced by therapeutic drugs [40]. Secondly, enhanced autophagy in SG-enriched tumors may facilitate the clearance of cytotoxic protein aggregates and damaged organelles because of mTOR inhibition, thus preserving intracellular metabolic homeostasis and promoting resistance [41]. Lastly, SGs might indirectly influence treatment outcomes through the modulation of immune-regulatory molecules within the TME [9]. In summary, we hypothesize that the predicted IC50 differences between risk groups may involve multiple pathways and that targeting stress granule-associated factors could be explored in future mechanistic studies. However, this hypothesis requires experimental validation in appropriate models.

The six prognostic genes identified in this study continue to hold potential for further investigation. SPAG5 is predominantly expressed in sperm cells and is involved in the regulation of cell division, playing a critical role particularly during mitosis [42]. The dysregulated expression of SPAG5 has been implicated in the initiation and progression of various tumors. A recent study showed that increased SPAG5 expression correlates with tumor proliferation and malignant transformation [43,44]. For example, SPAG5 could promote the proliferation of gastric cancer cells by regulating the PI3K/AKT signaling pathway [42]. SPAG5 expression was found to be significantly elevated in PCa samples, suggesting its possible involvement in the progression of PCa. And knocking out SPAG5 in prostate cancer cell lines significantly inhibited the proliferation, invasion, and migration of prostate cancer cells. These phenotypic changes are consistent with well-established hallmarks of cancer progression, whereby dysregulated proliferation, epithelial–mesenchymal transition (EMT), altered cell–matrix adhesion, and enhanced migratory and invasive capacities collectively fuel tumor metastatic dissemination. In prior experimental prostate cancer research, SPAG5 knockdown has similarly been shown to impair proliferation, colony formation, migration and invasion in PC-3-derived model systems, corroborating the oncogenic role of SPAG5 in prostate tumor cells. Such malignant cellular phenotypes are routinely interrogated via proliferation, colony formation, wound healing and Transwell invasion assays in pre-clinical oncology studies, providing a conceptual framework for interpreting the antitumor phenotypes observed upon SPAG5 knockdown in our experiments [45,46]. Likewise, EGF, another gene overexpressed in our PCa cohort, is a polypeptide secreted by various cell types interacting with the Epidermal Growth Factor Receptor (EGFR), thereby modulating cellular processes such as proliferation, differentiation, and migration [47,48]. Increasing evidence suggests that EGFR expression in PCa is associated with the heightened proliferative and invasive potential of cancer cells [49,50]. The normal prostate epithelium is not subjected to chronic hypoxia. However, it undergoes transient physiological stressors, including fluctuations in metabolic activity and mild oxidative stress, which may initiate the formation of small amounts of SGs [51]. In contrast, PCa develops within a persistently stressed environment characterized by hypoxia, nutrient deprivation, oxidative stress, and endoplasmic reticulum stress. Under conditions of sustained chronic stress, SGs accumulate. It is hypothesized that persistent SG formation might recruit and reduce the abundance of mRNAs encoding CCK, FN1, PTGS2, and SELE, which could be associated with tumor progression. Although the expression levels of these genes are very low in PCa samples, they still hold significant research value. Notably, FN1, a key extracellular matrix protein, is implicated in promoting PCa invasion and metastasis by facilitating cancer cell adhesion, migration, and the remodeling of the tumor microenvironment [52,53]. In contrast to its commonly reported protumorigenic role in the majority of solid tumors, our investigation revealed that FN1 expression is reduced in PCa specimens. This finding implies the existence of a distinct regulatory mechanism in PCa, warranting further confirmation through in vitro functional assays and validation across multicenter patient cohorts. Additionally, CCK, known primarily for stimulating gallbladder contraction to facilitate bile secretion and thereby contribute to lipid digestion and absorption, may also have significant functions in certain cancers and metabolic disorders, potentially exerting an indirect effect on the progression of PCa [54]. SELE facilitates the transport of white blood cells from the circulatory system to sites of inflammation. In the TME, it can promote the infiltration of antitumor immune cells. Conversely, it may also create an inflammatory microenvironment that promotes tumor growth [55]. PTGS2 (COX-2) regulates inflammatory and immune responses, vasodilation, and platelet aggregation. In environments with intense inflammatory activity, COX-2 primarily exhibits tumor-promoting effects; however, its precise role in the onset and progression of cancer requires further research for confirmation [56,57]. These genes offer valuable insights for mechanistic investigations and the development of targeted therapeutic strategies in PCa. However, the direct roles of these genes still need further experimental validation.

GSEA revealed significant enrichment in multiple pathways when comparing high- and low-risk groups. Among these, the arginine and proline metabolism pathway emerged as the most notable. Arginine and proline are crucial amino acids in the physiological system, integral to numerous metabolic pathways and playing a significant role in cancer metabolic reprogramming [58,59]. In PCa, arginine and proline metabolism have also been frequently reported. Research suggests that strategies modulating arginase activity to influence M2-to-M1-like macrophage polarization, regulate effector immune responses, and inhibit immune escape may reveal a useful therapeutic approach for PCa [59]. Immune infiltration analysis revealed markedly increased regulatory T cell (Treg) infiltration in high-risk PCa patients, with FN1 expression showing a strong positive association with Treg abundance (r = 0.60, *p* < 0.001). Tregs are an immunosuppressive T-cell subset that mediates immune tolerance and dampens the antitumor responses of effector T cells via the secretion of cytokines such as IL-10 and TGF-β [60,61]. In PCa, excessive Treg infiltration is closely associated with immune evasion and disease progression, as Tregs foster an immunosuppressive tumor microenvironment by dampening CD8+ T-cell function [61]. Aligning with the work of Ge et al., which defined two Treg-driven prostate cancer phenotypes—the immunosuppressive TregR (poor prognosis) and the less suppressive TregP (favorable outcome)—our HRG strongly corresponds to TregR, while the LRG matches TregP [62]. These observations highlight a close association between our high-risk subgroup and the Treg-driven immunosuppressive TregR phenotype. Future investigations are required to explore whether patients within this subgroup may respond to Treg-targeted therapeutic strategies.

The six-gene signature (SPAG5, FN1, CCK, EGF, SELE, PTGS2) demonstrates significant convergence with two established molecular frameworks in prostate cancer, thus validating its biological relevance. The HRG is congruent with both the immune-suppressed subtype as identified by Meng et al. and the PMOC2 classification [63,64]. This group is characterized by the increased infiltration of regulatory T cells (Tregs), as evidenced by a strong correlation between FN1 and Treg levels (r = 0.60); the upregulation of the immune checkpoint gene CD276, indicative of immune suppression; and the activation of cell cycle pathways, such as the G2/M checkpoint, which are associated with poor clinical outcomes. In contrast, the LRG corresponds to the immune-activated subtype identified by Meng et al. and the PMOC3 category, characterized by the elevated expression of CEACAM1, reduced Treg infiltration, and a balanced profile of oncogenic and inflammatory signaling pathways. The observed overlaps across different frameworks highlight the efficacy of the signature in integrating immune and multi-omics classifications. This provides a streamlined and reliable gene panel, which enhances risk stratification in prostate cancer.

The present study employed single-cell RNA sequencing (scRNA-seq) to validate the critical involvement of epithelial cells in carcinogenesis, as evidenced by their facilitation of prostate cancer invasion and mediation of pathogen responses [65,66]. In accordance with the observations reported by Zhu et al. [65], the present analysis identifies the presence of epithelial signatures, which are considered to be promising biomarkers for precision immunotherapy [67]. Pseudotime analysis revealed distinct temporal expression patterns, with FN1 and PTGS2 exhibiting upregulation during early differentiation stages, suggesting their potential involvement in facilitating initial cell migration and the establishment of an inflammatory TME. Conversely, CCK expression exhibited a marked increase during terminal differentiation, demonstrating a correlation with neuroendocrine characteristics and unfavorable clinical outcomes. These results underscore the pivotal role of temporal gene regulation along epithelial developmental pathways in driving PCa heterogeneity and disease progression.

## 5. Conclusions

This study utilized multi-omics data to develop a prostate cancer risk model based on six prognostic genes screened from the stress granule-related gene set, revealing their roles in epithelial differentiation and immune-related intercellular communication. Although potential therapeutic targets were identified, several limitations should be acknowledged. Firstly, the dependence on publicly available databases necessitates validation in independent prospective cohorts. Secondly, due to experimental constraints, SG induction and co-localization assays were not performed, and the in vitro functional experiments largely reproduced previously reported phenotypes of SPAG5 knockdown in prostate cancer cells, leaving the direct causal relationship between SGs and the prognostic genes unestablished. And we could not experimentally test whether SPAG5 modulates stress granule assembly under sodium arsenite, hypoxia or androgen deprivation stress. Our designation of these genes as “SG-derived” reflects their origin from the GeneCards SG-related collection and subsequent prognostic screening, rather than experimental evidence of functional interaction with SGs. The biological connection between these genes and stress granules remains speculative and needs to be addressed in future independent investigations. Thirdly, the AUC values of the risk model ranged from 0.60 to 0.76 across different time points, indicating only moderate predictive accuracy; therefore, the model should be interpreted as an adjunct to conventional clinicopathological parameters rather than a standalone decision tool, and further external validation in larger, multicenter prospective cohorts is necessary to confirm its clinical utility. Fourthly, epithelial cells in the scRNA-seq analysis were annotated based on canonical marker genes as in the original GSE176031 study, which described them as “tumor-associated epithelial cells” rather than definitively malignant cells. We did not perform copy number variation-based methods (e.g., inferCNV or CopyKAT) to verify their malignant status. Therefore, our single-cell results are primarily used for the descriptive characterization of cell type composition and expression patterns, rather than as definitive evidence of malignancy. Fifthly, the IHC validation was limited to representative images from five paired samples without quantitative scoring or independent pathological assessment. Therefore, no conclusion regarding the association between protein expression and clinicopathological features or prognosis can be drawn from these data, and larger cohorts with standardized scoring criteria are needed. Sixthly, the functional experiments were performed using PC-3 and 22Rv1 cell lines, which represent advanced/metastatic prostate cancer, whereas the prognostic risk model was developed from TCGA-PRAD localized primary tumors; thus, further validation in early-stage models is warranted. Future investigations will focus on confirming this regulatory mechanism, validating the risk model through multicenter prospective cohorts, and elucidating the causal functions of SPAG5 and FN1 using CRISPR technology or patient-derived xenograft (PDX) models to assess their translational potential.

## Figures and Tables

**Figure 1:**
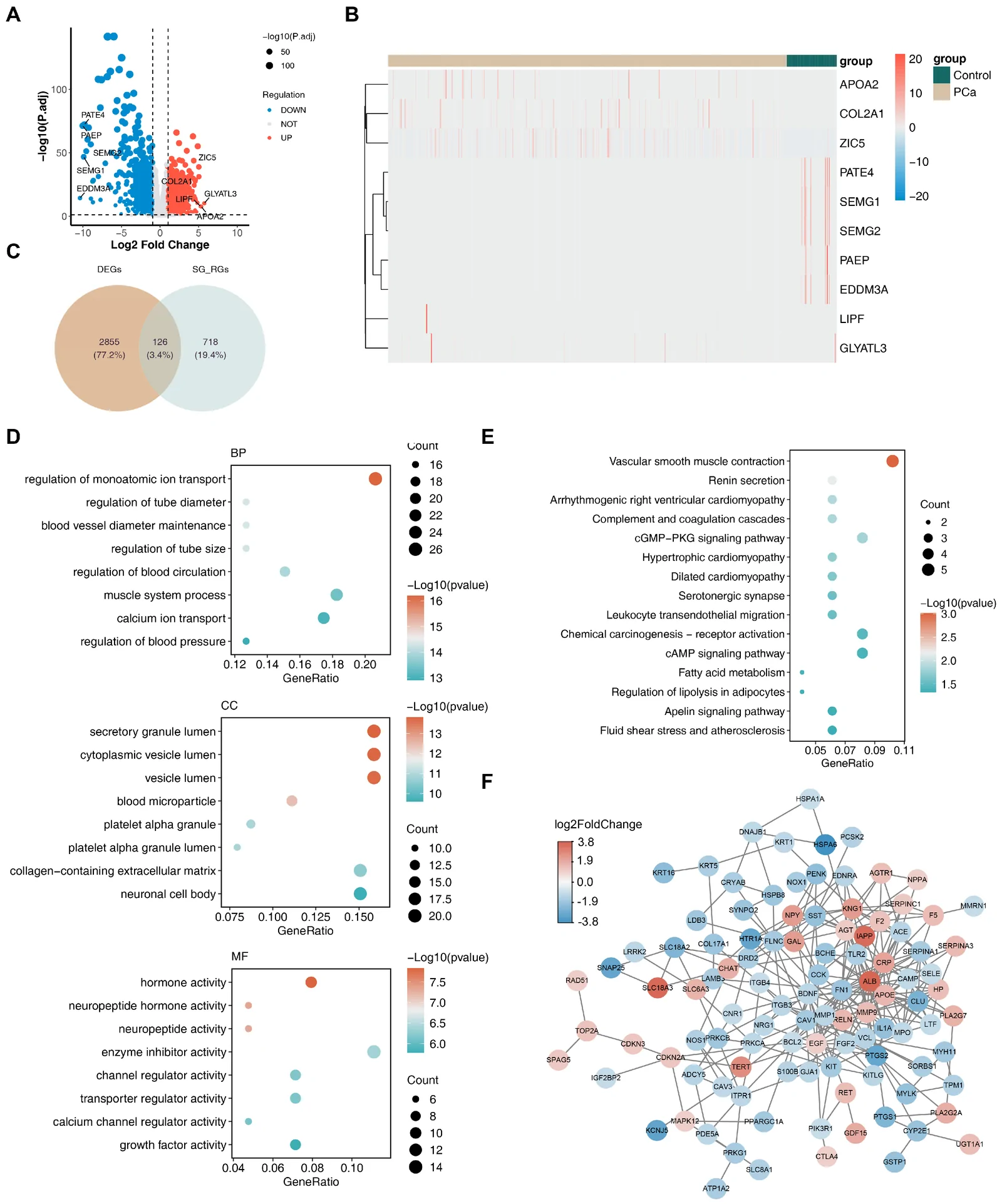
Identification of 126 candidate stress granule-related genes and their functional characteristics in prostate cancer. (**A**,**B**) Differential expression analysis reveals genes significantly altered in prostate cancer. (**C**) Intersection of DEGs and SG-RGs defines candidate gene set. (**D**,**E**) GO and KEGG analyses reveal that candidate genes are involved in ion transport, secretory granules, hormone activity, and pathways like vascular smooth muscle contraction and complement cascades. (**F**) PPI network highlights ALB as potential hub gene interacting with F2, CRP, and AGT.

**Figure 2:**
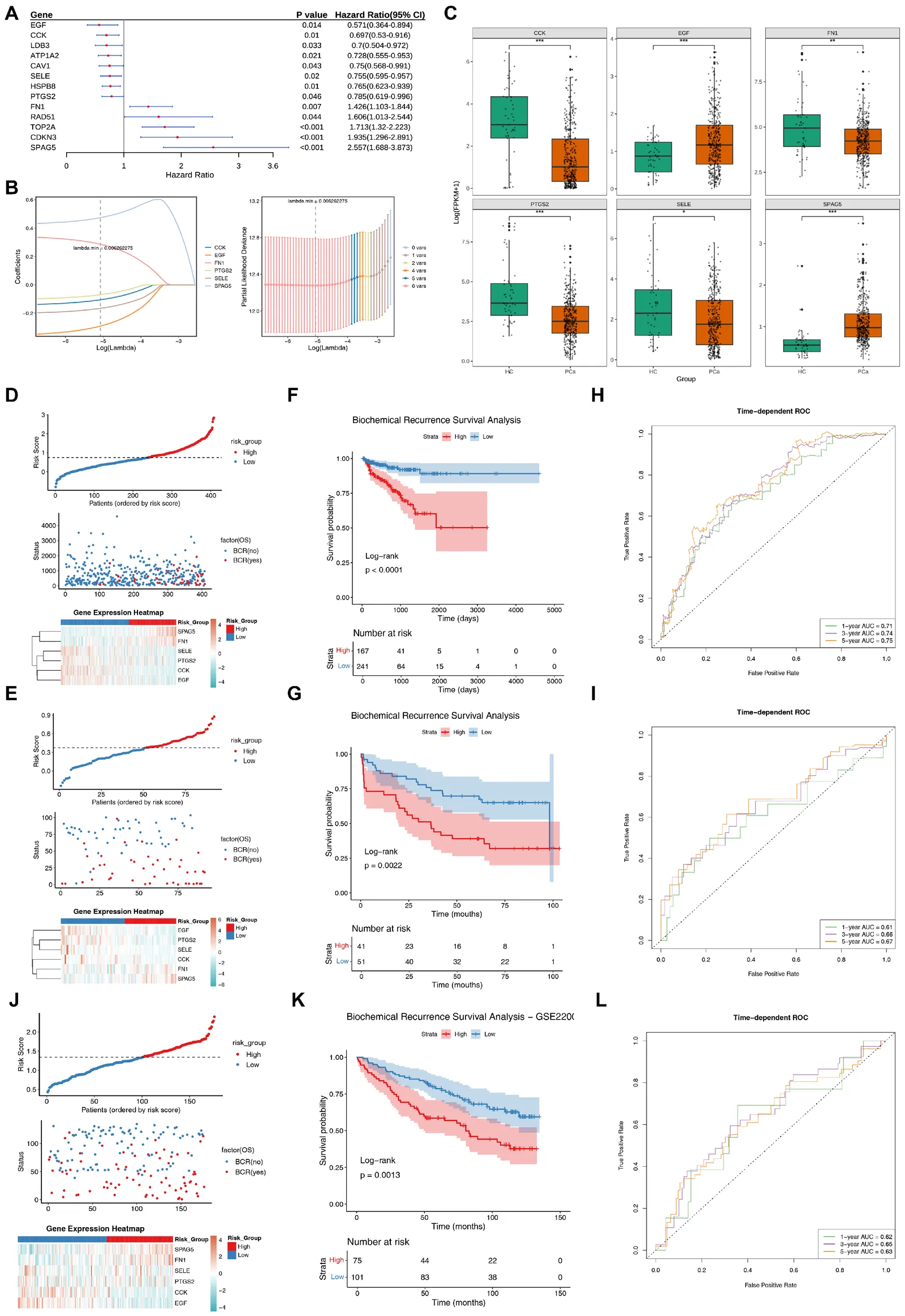
Development and assessment of six-gene prognostic signature (GSE70769 and GSE220095). (**A**,**B**) Univariate Cox and LASSO regression identify six prognostic genes (SPAG5, FN1, CCK, EGF, SELE, PTGS2). * *p* < 0.05, ** *p* < 0.01, *** *p* < 0.001. (**C**) Validation of differential expression for six genes. (**D**,**E**,**J**) Patients stratified into HRG and LRG show distinct risk score distributions, survival outcomes, and gene expression patterns in both training and validation sets. (**F**,**G**,**K**) HRG patients have significantly worse BCR-free survival. (**H**,**I**,**L**) Risk model demonstrates robust predictive performance over 1, 3, and 5 years.

**Figure 3:**
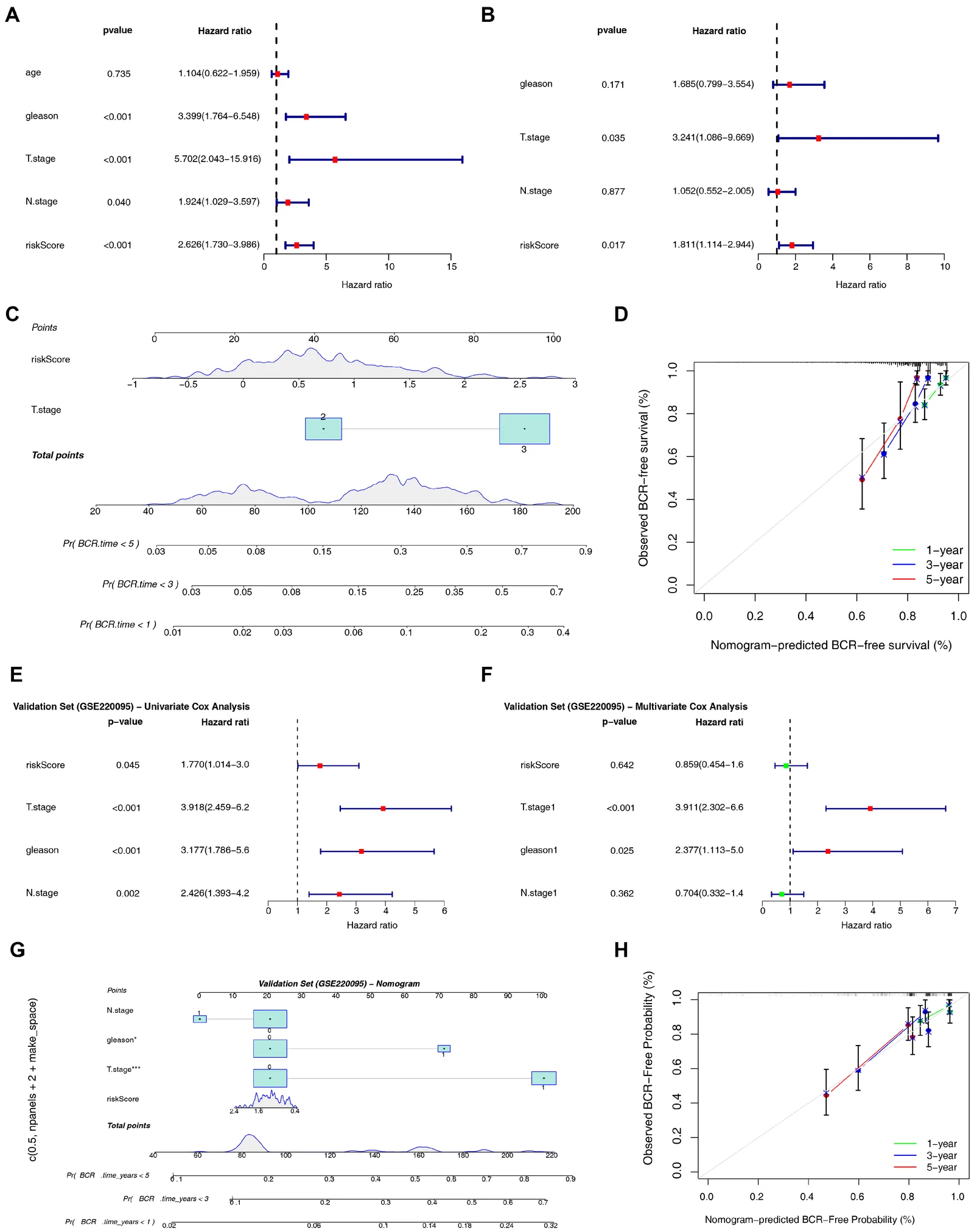
Establishment of nomogram for individualized prognosis prediction (GSE70769 and GSE220095). (**A**,**B**,**E**,**F**) Univariate and multivariate Cox analyses identify risk score and T stage as independent prognostic factors. (**C**,**G**) Nomogram allows for calculation of total point score to estimate survival probability. (**D**,**H**) Calibration curves show good agreement between nomogram-predicted survival and actual observed survival. * *p* < 0.05, *** *p* < 0.00.

**Figure 4:**
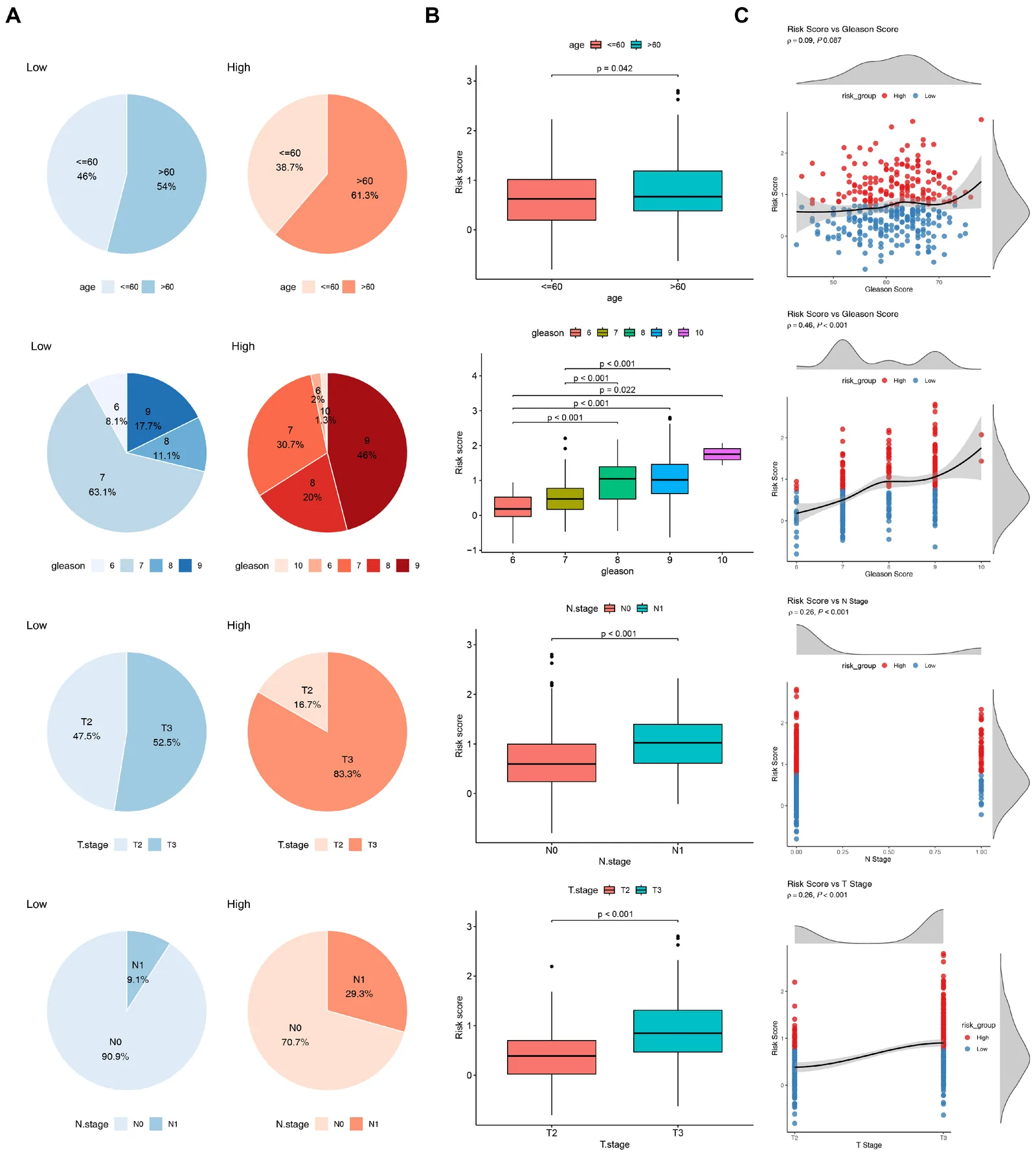
Correlation of risk model with clinicopathological features. (**A**) Proportion of patients with different clinical characteristics in HRG and LRG. (**B**) Risk scores are significantly higher in patients with advanced age (>60), positive nodal status (N1), advanced tumor stage (T3), and higher Gleason scores. (**C**) Significant positive correlation exists between risk score and Gleason score.

**Figure 5:**
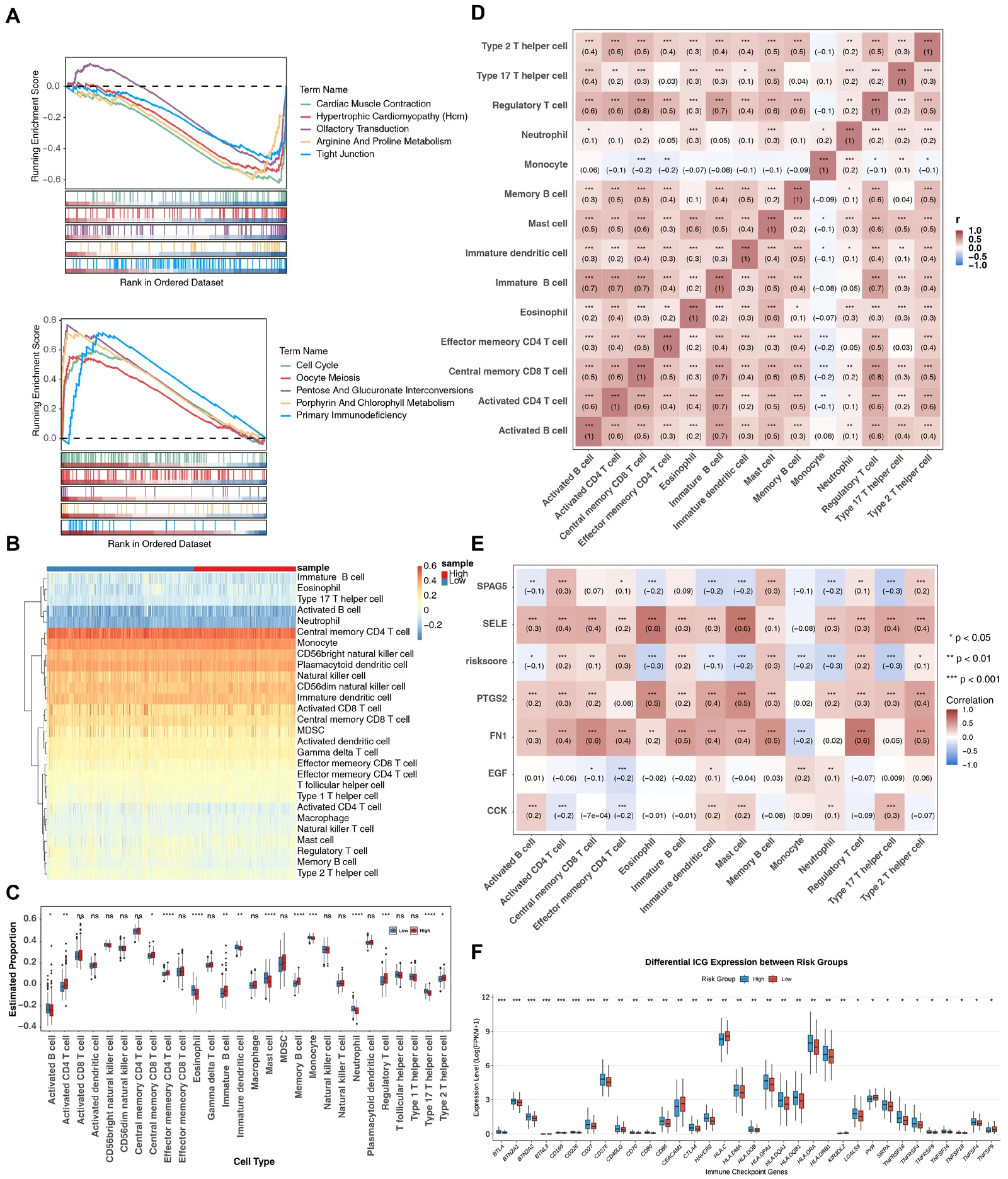
Comprehensive characterization of biological pathways and immune landscape. (**A**) GSEA analysis based on KEGG pathway gene set (c2.cp.kegg.v2022.1.Hs.symbols.gmt) reveals enrichment in cell cycle pathways in HRG (upper panel) and metabolic/structural pathways in LRG (lower panel). (**B**,**C**) Immune infiltration analysis identifies 14 immune cell types with significant abundance differences between groups. * *p* < 0.05, ** *p* < 0.01, *** *p* < 0.001, **** *p* < 0.0001. (**D**,**E**) Correlation analyses reveal interconnections among immune cells and between prognostic genes and immune cells. (**F**) Differential expression of ICGs, such as elevated CD276 in HRG and CEACAM1 in LRG, suggests distinct immune evasion mechanisms.

**Figure 6:**
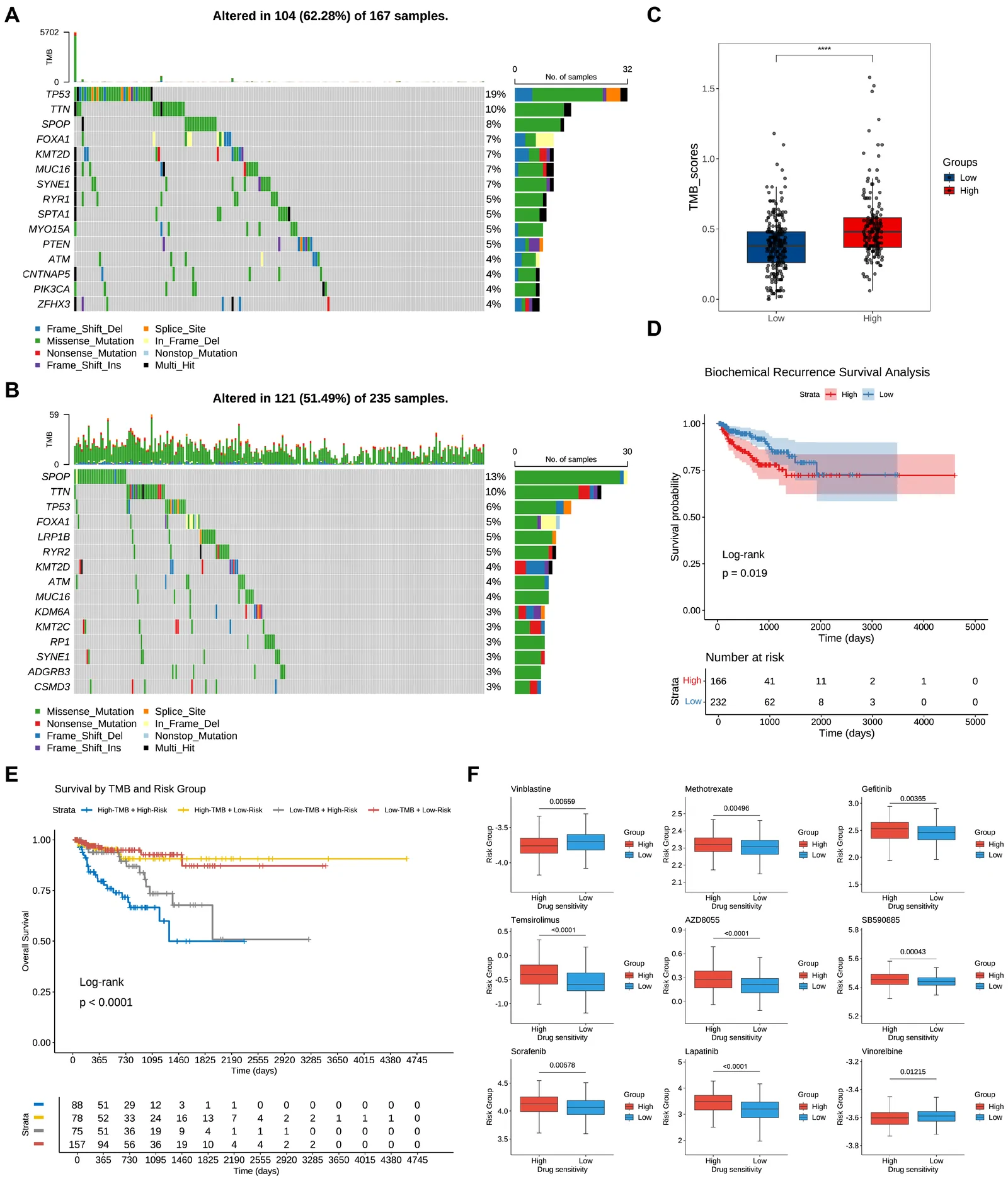
Genomic alterations and therapeutic implications. (**A**,**B**) Somatic mutation profiles show high frequencies of TP53, TTN, and SPOP mutations in both groups. (**C**) HRG patients have significantly higher TMB. **** *p* < 0.0001. (**D**,**E**) Survival analysis confirms poorer prognosis for patients with high TMB and, particularly, high-TMB + HRG subgroup. (**F**) Drug sensitivity analysis reveals differential responses, e.g., HRG patients are less sensitive to temsirolimus.

**Figure 7:**
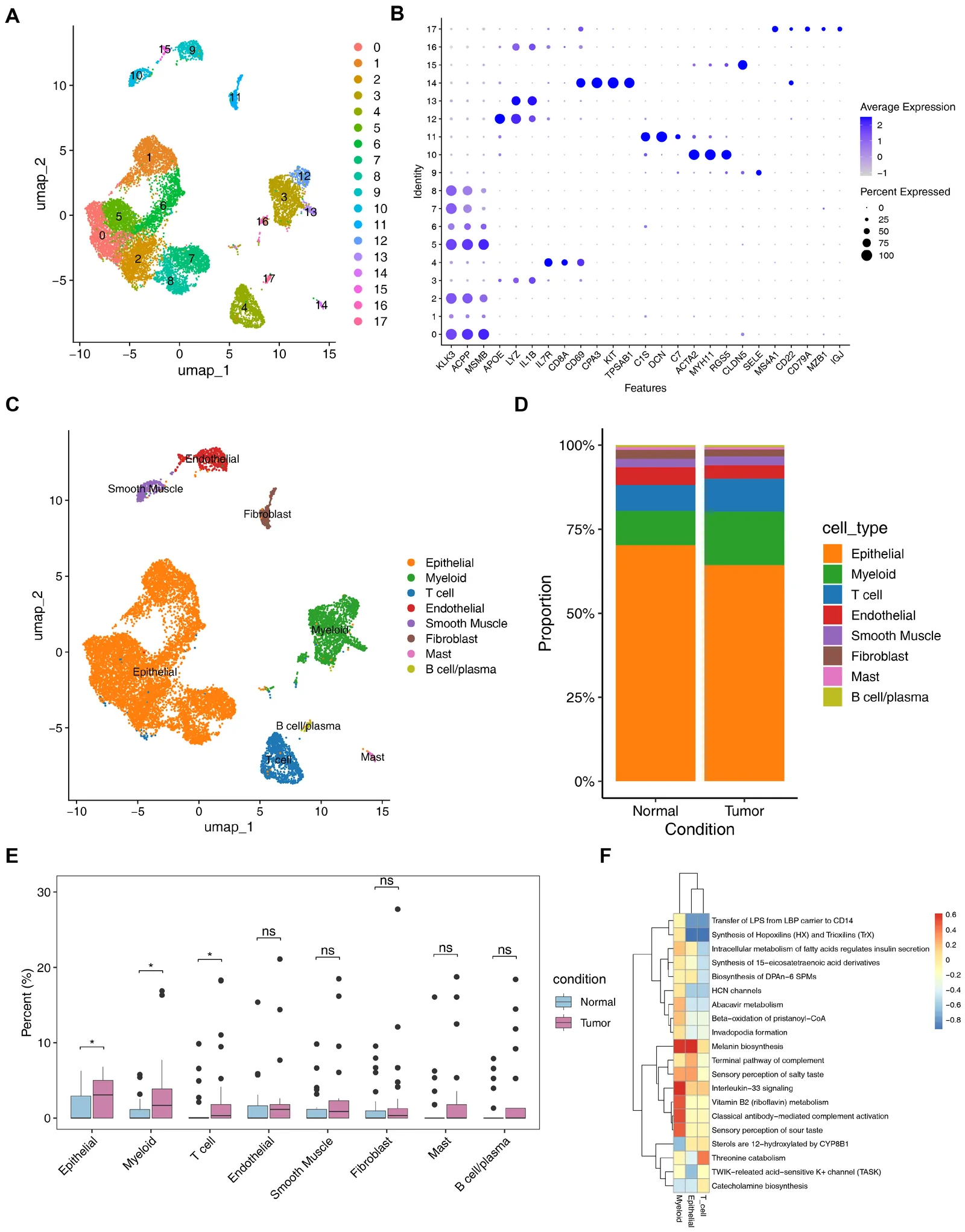
Cellular atlas of prostate tumor microenvironment. (**A**–**C**) Unsupervised clustering and annotation identify major cell types, including epithelial, T, myeloid, and B cells. (**D**,**E**) Epithelial, myeloid, and T cells show significant abundance differences between tumor and normal tissues. * *p* < 0.05. (**F**) Differentially abundant cells are enriched in pathways like “melanin biosynthesis”.

**Figure 8:**
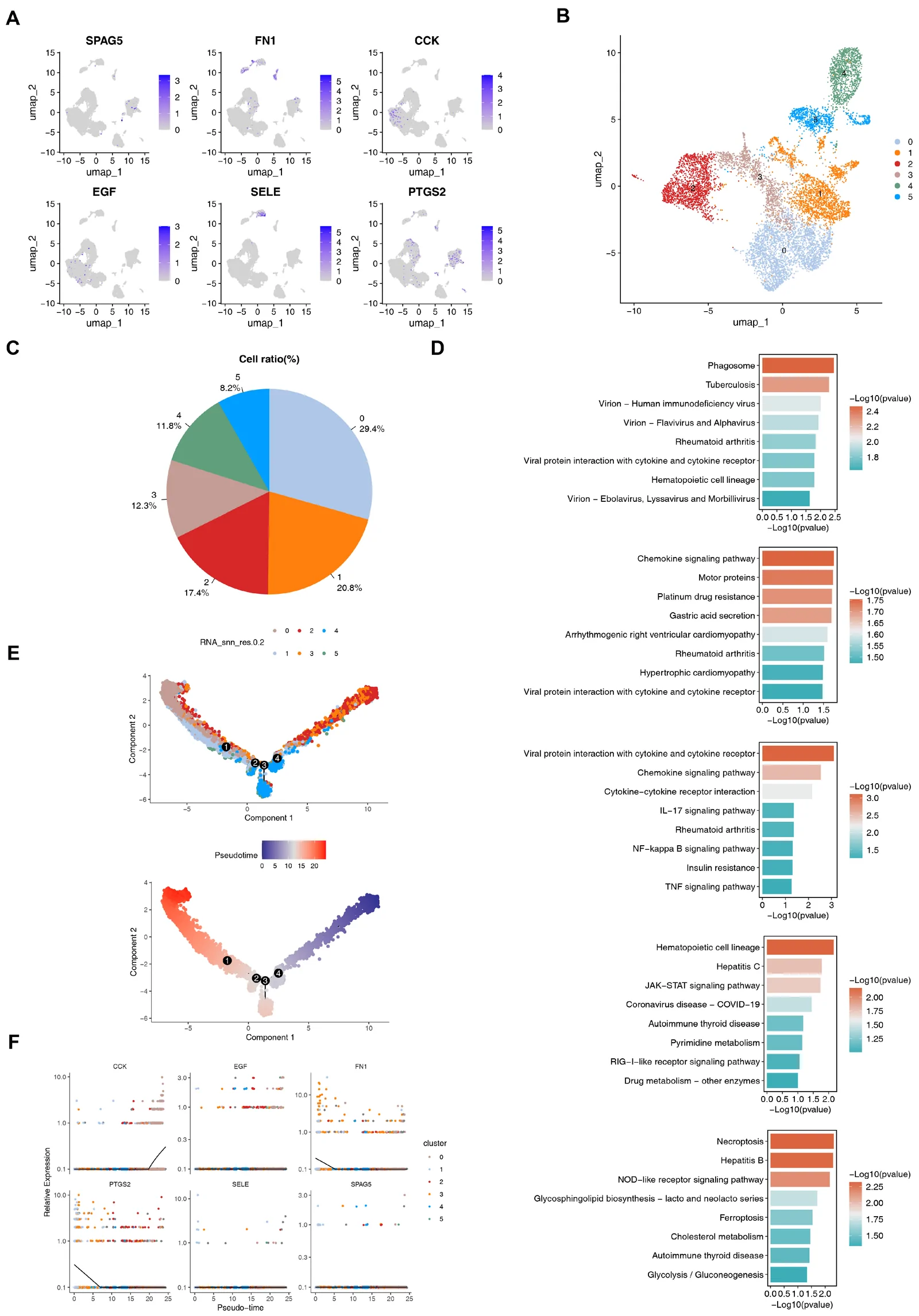
Deep dive into epithelial cell heterogeneity. (**A**) Prognostic genes show varied expression across cell types; CCK is significantly downregulated in tumor epithelial cells. (**B**,**C**) Epithelial cells are subdivided into 6 subclusters, with Subcluster 0 being most prevalent. (**D**) Subclusters are associated with distinct pathways. (**E**,**F**) Pseudotime analysis reveals differentiation trajectory with dynamic gene expression; FN1 and PTGS2 are highly expressed early, while CCK increases later.

**Figure 9:**
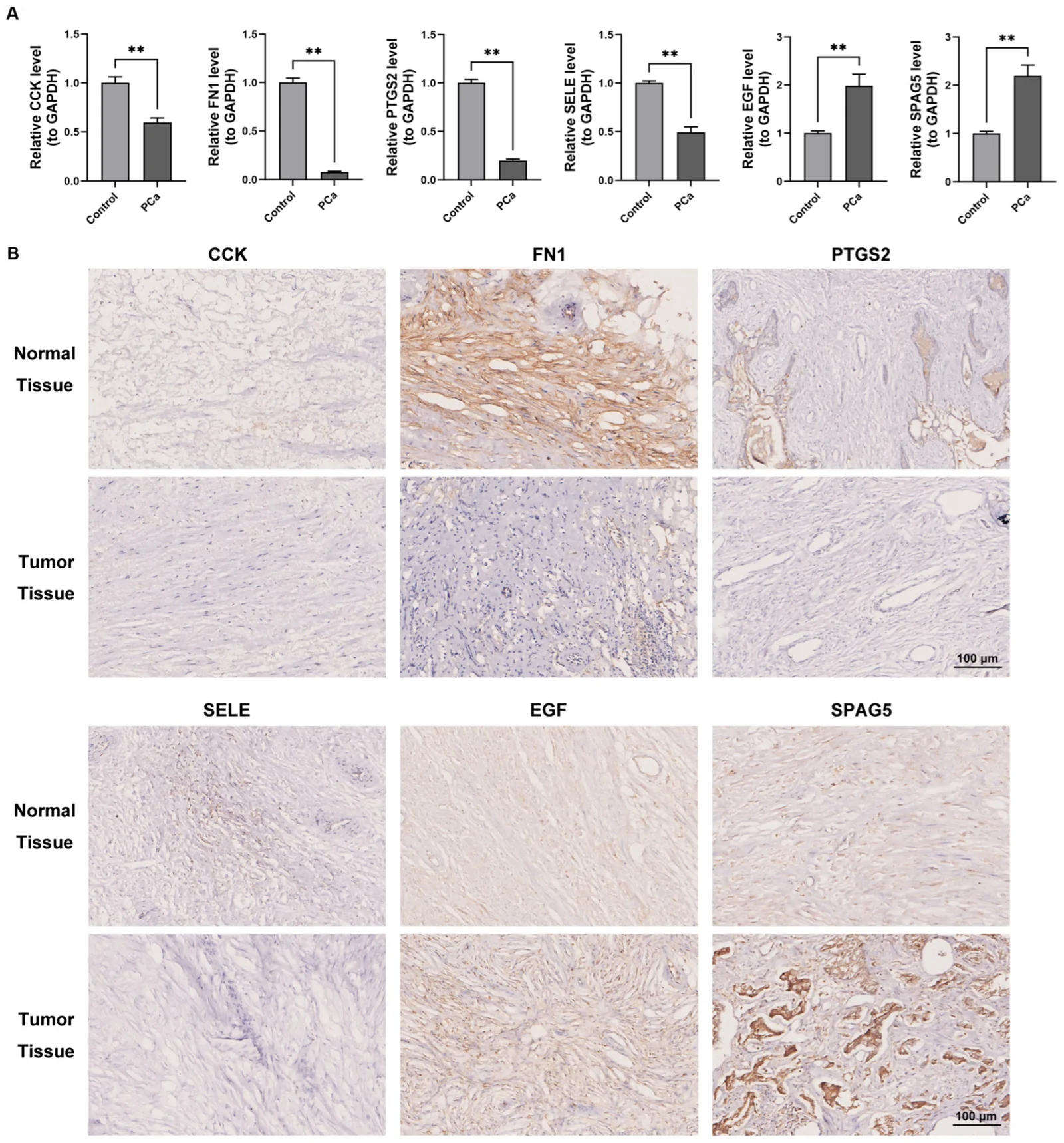
Validation of prognostic gene expression in prostate cancer tissues. (**A**) Validation of prognostic gene expression in prostate cancer tissues by RT-qPCR. ** *p* < 0.01. (**B**) Immunohistochemical validation of prognostic protein expression in prostate cancer and adjacent normal tissues.

**Figure 10:**
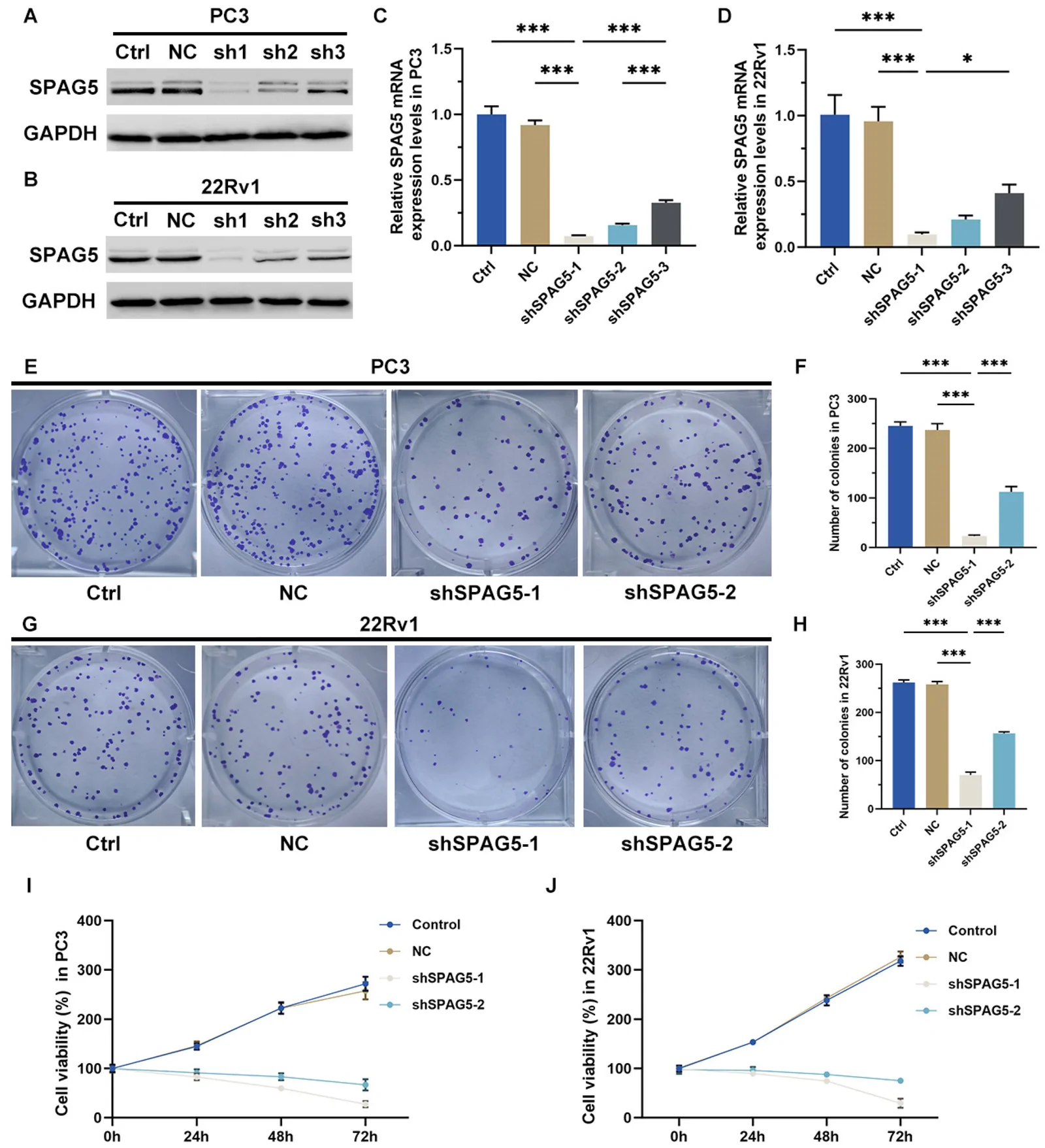
SPAG5 knockdown inhibits proliferation, migration, and invasion of prostate cancer cells in vitro. (**A**,**B**) Western blot analysis of SPAG5 protein expression in PC-3 (**A**) and 22Rv1 (**B**) cells after transduction with three independent shRNAs targeting SPAG5 (sh1, sh2, sh3) or negative control (NC), with Ctrl representing untreated cells. GAPDH was used as loading control. (**C**,**D**) RT-qPCR quantification of SPAG5 mRNA levels in PC-3 (**C**) and 22Rv1 (**D**) cells following shRNA-mediated knockdown, *** *p* < 0.001. (**E**,**F**) Representative images (**E**) and quantitative analysis (**F**) of colony formation assays in PC-3 cells. (**G**,**H**) Representative images (**G**) and quantitative analysis (**H**) of colony formation assays in 22Rv1 cells, *** *p* < 0.001. (**I**,**J**) CCK-8 assays assessing relative cell growth at 0, 24, 48, and 72 h in PC-3 (**I**) and 22Rv1 (**J**) cells after SPAG5 knockdown. * *p* < 0.05, *** *p* < 0.001.

**Figure 11:**
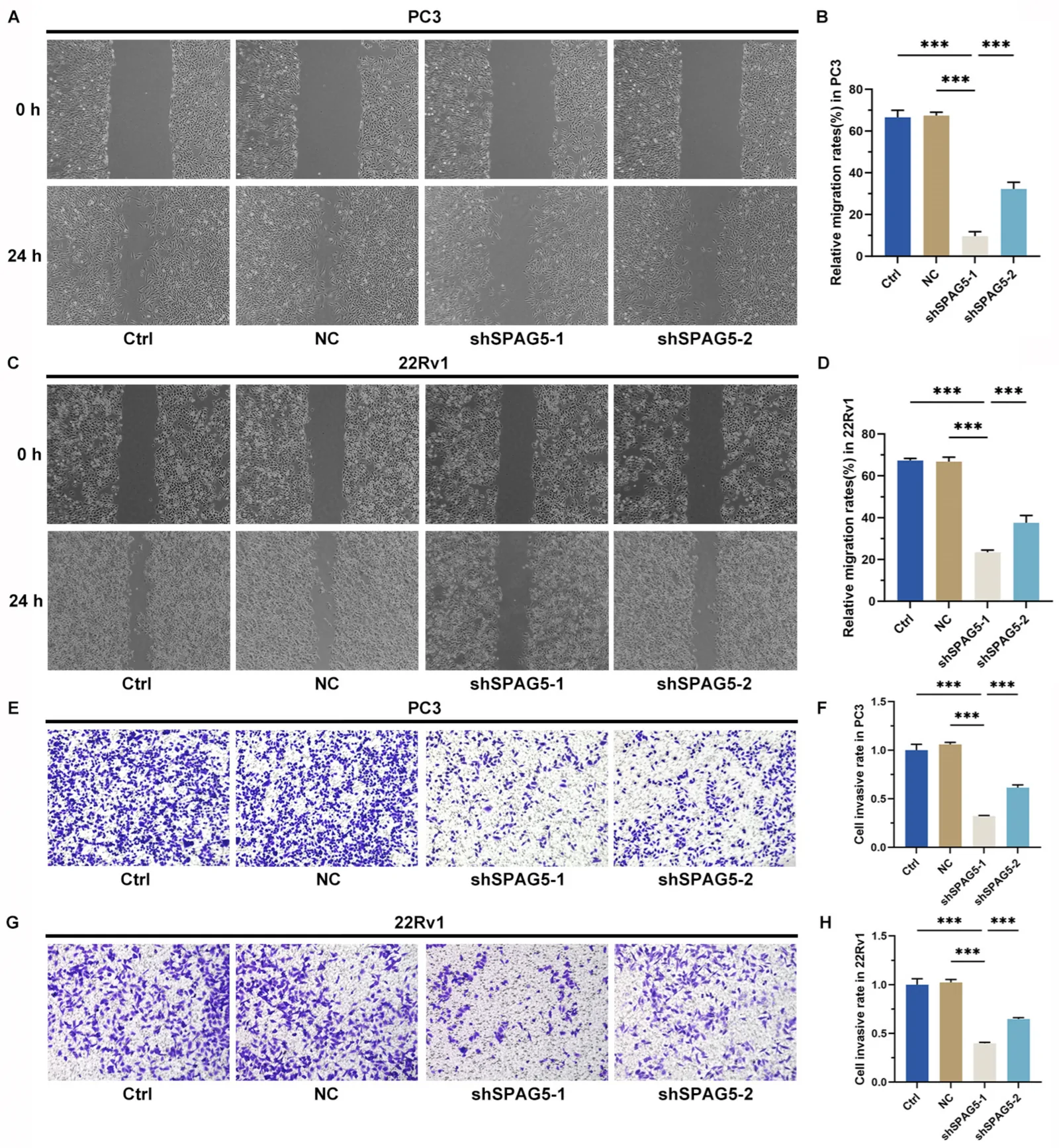
SPAG5 knockdown suppresses migration and invasion of prostate cancer cells. (**A**,**B**) Representative images (**A**) and quantitative analysis (**B**) of wound healing assays in PC-3 cells at 0 h and 24 h post-scratching. (**C**,**D**) Representative images (**C**) and quantitative analysis (**D**) of wound healing assays in 22Rv1 cells. (**E**,**F**) Representative images (**E**) and quantitative analysis (**F**) of Transwell invasion assays in PC-3 cells. (**G**,**H**) Representative images (**G**) and quantitative analysis (**H**) of Transwell invasion assays in 22Rv1 cells. Cells after transduction with two independent shRNAs targeting SPAG5 (sh1, sh2) or negative control (NC), with Ctrl representing untreated cells. *** *p* < 0.001 vs. Ctrl or NC.

## Data Availability

The transcriptomic, survival, and single-cell RNA sequencing data used in this study were obtained from the TCGA database (https://portal.gdc.cancer.gov/, reference: TCGA-PRAD, accessed on 31 July 2025) and the GEO database (https://www.ncbi.nlm.nih.gov/geo/, accessed on 31 July 2025), specifically including the GSE70769 and GSE176031 datasets. The relevant data are available from the corresponding author upon reasonable request.

## Supplementary Materials

The following supporting information can be downloaded at https://www.mdpi.com/article/10.3390/biomedicines14102165/s1, Supplementary Figure S1: PH assumption test for univariate cox regression. Supplementary Figure S2: The corrected immune infiltration results based on the PH hypothesis test for the multivariate Cox regression model. Supplementary Figure S3: QC and feature selection for single-cell RNA sequencing data. (A) QC plots showing the distribution of detected genes per cell (nFeature_RNA), total counts per cell (nCount_RNA), and mitochondrial gene percentage (percent.mt) before and after filtering. Supplementary Figure S4: Expression analysis of prognostic genes in differentially abundant cell types. Supplementary Table S1: List of stress granule-related genes. Supplementary Table S2: BCR event counts and Harrell’s C-index of the risk model across cohorts. Supplementary Table S3: Incremental prognostic value of risk score over Gleason score across cohorts. Supplementary Table S4: Enriched KEGG pathways in high- and low-risk groups identified by GSEA.

## Abbreviations

The following abbreviations are used in this manuscript:

ADT	Androgen Deprivation Therapy
AUC	Area Under the Curve
BCR	Biochemical Recurrence
cDNA	Complementary DNA
CRPC	Castration-Resistant Prostate Cancer
DEGs	Differentially Expressed Genes
EGF	Epidermal Growth Factor
EGFR	Epidermal Growth Factor Receptor
FC	Fold Change
FDR	False Discovery Rate
GEO	Gene Expression Omnibus
GO	Gene Ontology
GSEA	Gene Set Enrichment Analysis
HRG	High-Risk Group
HVGs	Highly Variable Genes
IHC	Immunohistochemistry
ICGs	Immune Checkpoint Genes
KEGG	Kyoto Encyclopedia of Genes and Genomes
LASSO	Least Absolute Shrinkage and Selection Operator
LLPS	Liquid–Liquid Phase Separation
LRG	Low-Risk Group
mCRPC	Metastatic Castration-Resistant Prostate Cancer
mTOR	Mechanistic Target of Rapamycin
PCA	Principal Component Analysis
PCa	Prostate Cancer
PH	Proportional Hazards
PPI	Protein–Protein Interaction
QC	Quality Control
ROC	Receiver Operating Characteristic
RT-qPCR	Reverse Transcription Quantitative Polymerase Chain Reaction
scRNA-seq	Single-Cell RNA Sequencing
SGs	Stress Granules
SG-RGs	Stress Granule-Related Genes
TCGA	The Cancer Genome Atlas
TCGA-PRAD	The Cancer Genome Atlas Prostate Adenocarcinoma
TMB	Tumor Mutational Burden
Tregs	Regulatory T Cells
UMAP	Uniform Manifold Approximation and Projection
