# Supplementary material for: A Prognostic Model for Prostate Cancer Screened from Stress Granule-Related Genes: An Integrated Bioinformatic Analysis and Experimental Verification of SPAG5 Function
Source: Biomedicines. 2026 Sep 24;14(10):2165. doi: 10.3390/biomedicines14102165 (PMC13701839; doi:10.3390/biomedicines14102165)
Supplement: Supplementary file 1 [file biomedicines-14-02165-s001.zip › Supplementary Figures S1-S4.pdf]

Supplementary Figures

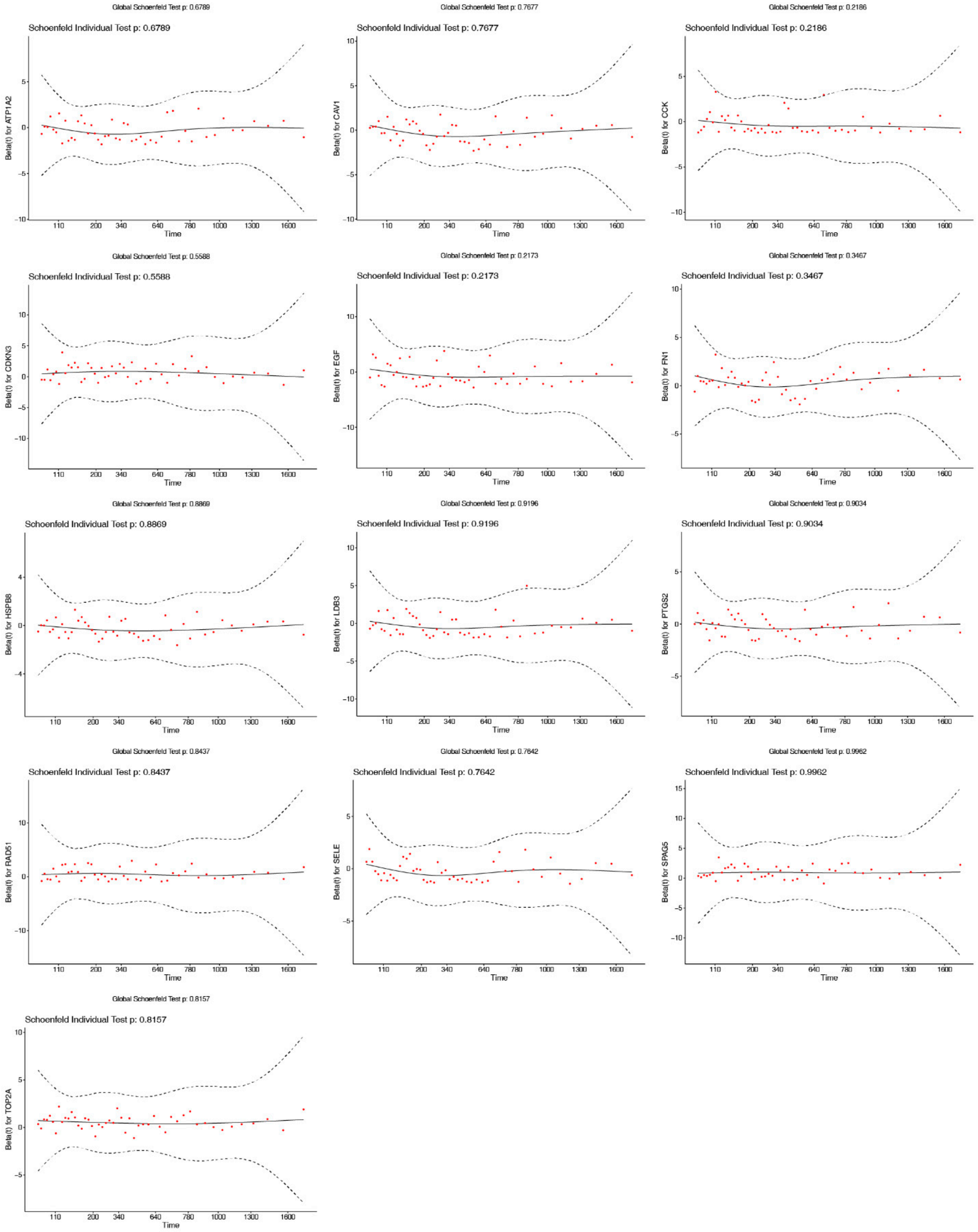

Figure S1. PH assumption test for univariate cox regression.

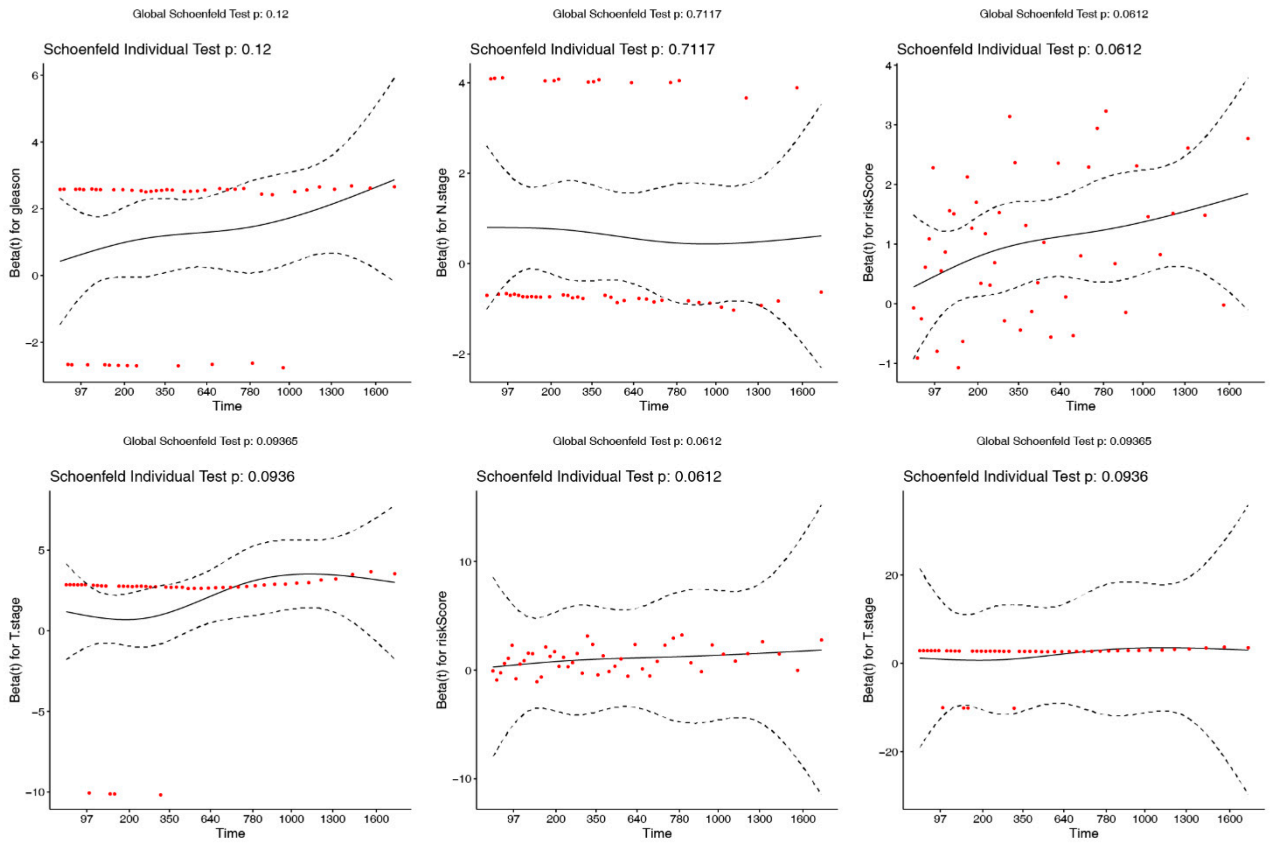

Figure S2. The corrected immune infiltration results based on the PH hypothesis test for the multivariate Cox regression model.

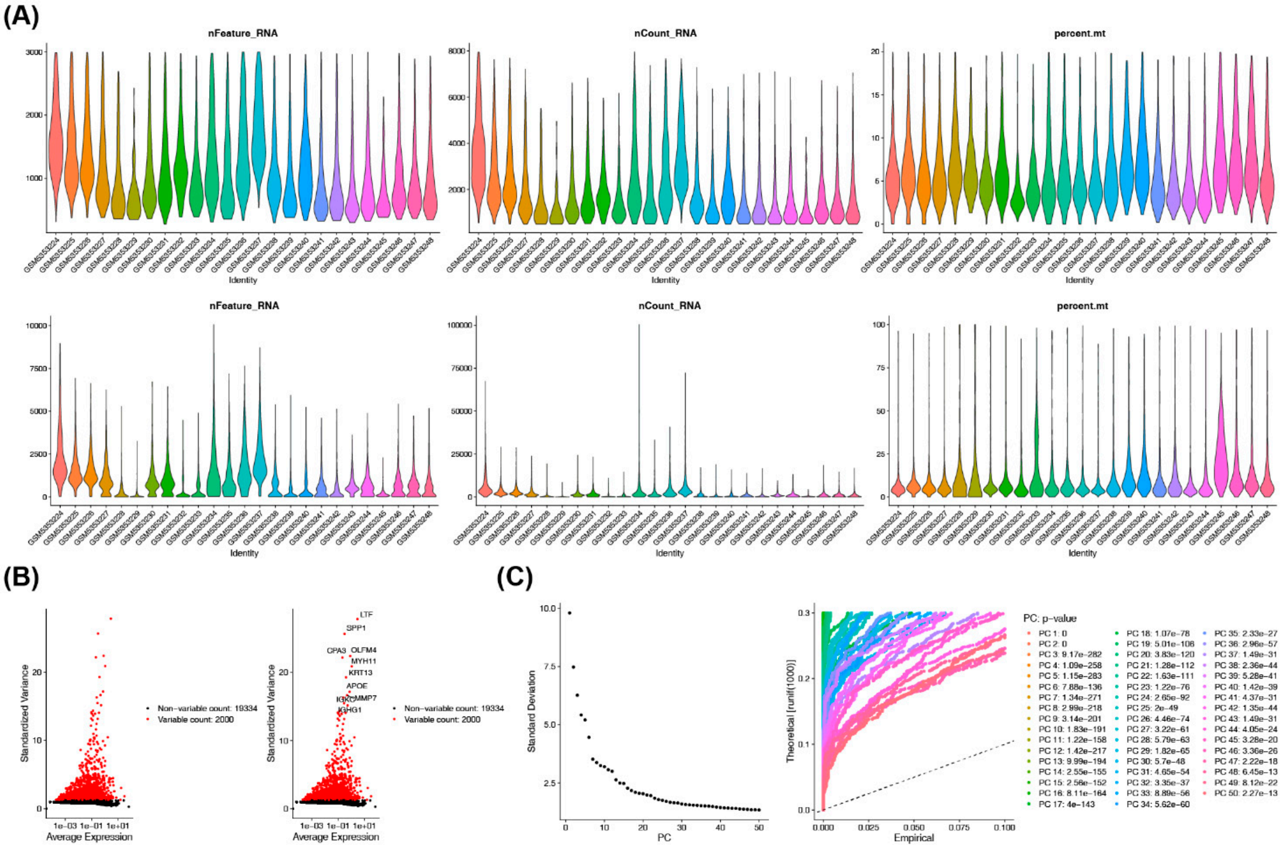

Figure S3. QC and feature selection for single-cell RNA sequencing data. (A) QC plots showing the distribution of detected genes per cell (nFeature_RNA), total counts per cell (nCount_RNA), and mitochondrial gene percentage (percent.mt) before and after filtering.

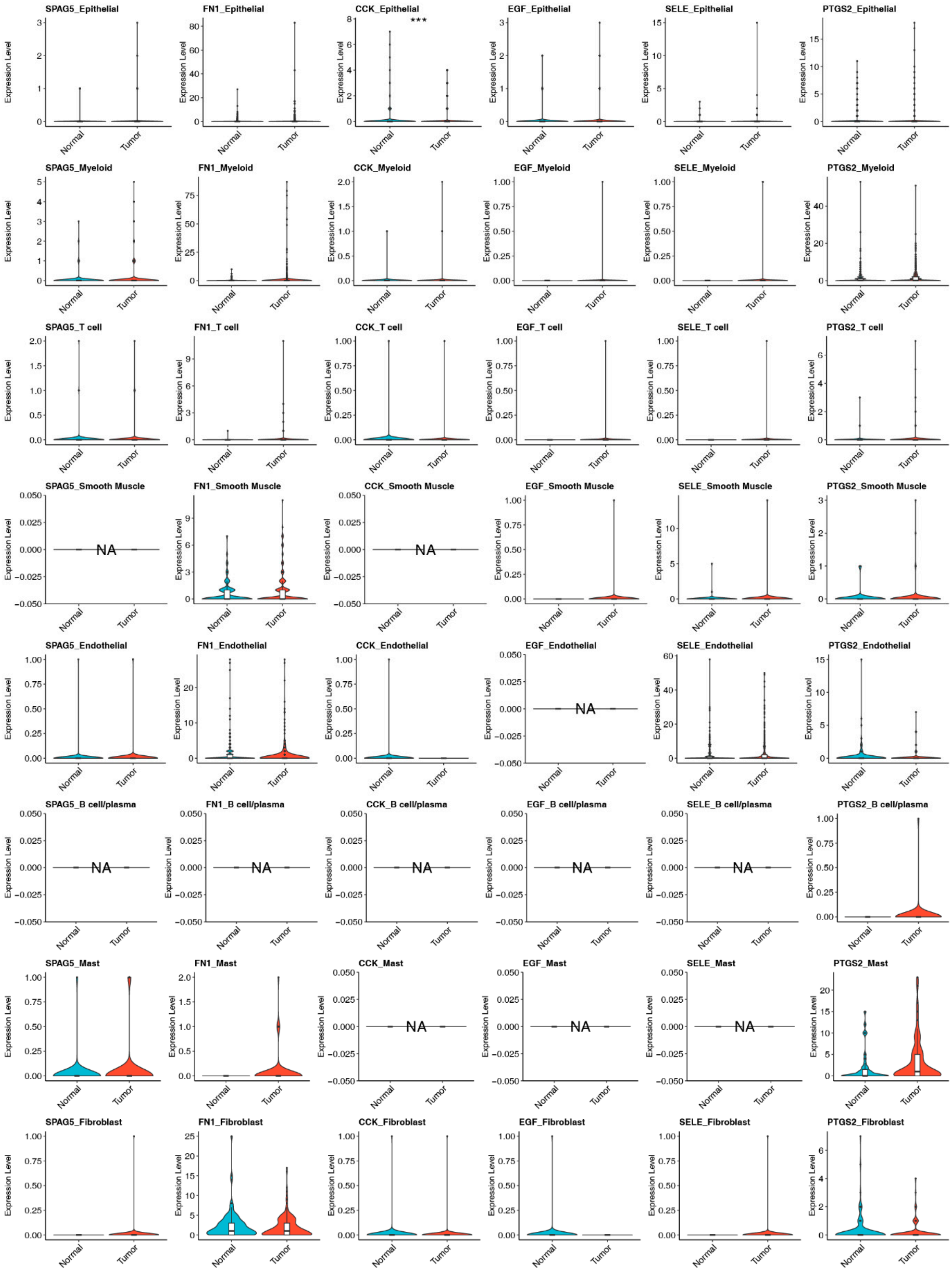

Figure S4. Expression analysis of prognostic genes in differentially abundant cell types.
